# Characterizing microRNA editing and mutation sites in Autism Spectrum Disorder

**DOI:** 10.3389/fnmol.2022.1105278

**Published:** 2023-01-20

**Authors:** Xingwang Wu, Huaide Yang, Han Lin, Angbaji Suo, Shuai Wu, Wenping Xie, Nan Zhou, Shiyong Guo, Hao Ding, Guangchen Zhou, Zhichao Qiu, Hong Shi, Jun Yang, Yun Zheng

**Affiliations:** ^1^State Key Laboratory of Primate Biomedical Research, Kunming University of Science and Technology, Kunming, Yunnan, China; ^2^Institute of Primate Translational Medicine, Kunming University of Science and Technology, Kunming, Yunnan, China; ^3^Faculty of Information Engineering and Automation, Kunming University of Science and Technology, Kunming, Yunnan, China; ^4^Department of Urology, The First Affiliated Hospital of Kunming Medical University, Kunming, Yunnan, China; ^5^School of Criminal Investigation, Yunnan Police College, Kunming, Yunnan, China; ^6^College of Landscape and Horticulture, Yunnan Agricultural University, Kunming, Yunnan, China

**Keywords:** miRNA editing, Autism Spectrum Disorder, hsa-miR-376a-5p, GPR85, NAPB

## Abstract

Autism Spectrum Disorder (ASD) is a neurodevelopmental disorder whose pathogenesis is still unclear. MicroRNAs (miRNAs) are a kind of endogenous small non-coding RNAs that play important roles in the post-transcriptional regulation of genes. Recent researches show that miRNAs are edited in multiple ways especially in central nervous systems. A-to-I editing of RNA catalyzed by Adenosine deaminases acting on RNA (ADARs) happens intensively in brain and is also noticed in other organs and tissues. Although miRNAs are widely edited in human brain, miRNA editing in ASD is still largely unexplored. In order to reveal the editing events of miRNAs in ASD, we analyzed 131 miRNA-seq samples from 8 different brain regions of ASD patients and normal controls. We identified 834 editing sites with significant editing levels, of which 70 sites showed significantly different editing levels in the superior frontal gyrus samples of ASD patients (ASD-SFG) when compared with those of control samples. The editing level of an A-to-I editing site in hsa-mir-376a-1 (hsa-mir-376a-1_9_A_g) in ASD-SFG is higher than that of normal controls, and the difference is exaggerated in individuals under 10 years. The increased expression of *ADAR1* is consistent with the increased editing level of hsa-mir-376a-1_9_A_g in ASD-SFG samples compared to normal SFG samples. Furthermore, we verify that A-to-I edited hsa-mir-376a-5p directly represses *GPR85* and *NAPB*, which may contribute to the abnormal neuronal development of ASD patients. These results provide new insights into the mechanism of ASD.

## 1. Introduction

Autism Spectrum Disorder (ASD) is a complex neurodevelopmental disorder, which occurs in early childhood (Baxter et al., 2015; Christensen et al., 2019). There are three basic characteristics of autism: interpersonal communication disorder, language communication disorder, and behavior stereotyping (Kanner, 1943; Minshew and Williams, 2007; Lai et al., 2013). ASD mainly starts before the age of 3, and the most obvious stage of ASD behavior is 2–5 years old. The exact cause of ASD is still unknown.

MicroRNAs (miRNAs) are a class of small non-coding RNAs of 21–22 nucleotides in length that mainly repress their target mRNAs at post-transcriptional level (Bartel, 2004). MiRNAs are involved in biological processes such as cell cycle, differentiation, development, and metabolism (Small and Olson, 2011; Khach Lai et al., 2012; Ng et al., 2012; Rottiers and Näär, 2012; Tong et al., 2012; Trompeter et al., 2013). It has been reported that miRNAs play important roles in various diseases (Esquela-Kerscher and Slack, 2006), including, but not limited to, breast cancer (Yan et al., 2008; Loh et al., 2019), liver cancer (Wang et al., 2014; Callegari et al., 2015), colon cancer (Valeri et al., 2014; Mohammadi et al., 2016), lung cancer (Yanaihara et al., 2006; Iqbal et al., 2019; Zhong et al., 2021), cardiovascular disease (Olson et al., 2012), Parkinson's disease (Leggio et al., 2017; Goh et al., 2019), Alzheimer's disease (Cogswell et al., 2008; Delay et al., 2012), and diabetes (Kantharidis et al., 2011). MiRNAs are also involved in ASD. For examples, the mitogen-activated protein kinases (MAPK) signaling pathway, in which the candidate target genes of both let-7a and let-7d are involved, is directly or indirectly associated with the physiopathology of ASD (Huang et al., 2015). The overexpression of miR-21-3p leads to a significant decrease in the expression of protocadherin 19 (*PCDH19*), which is related to cognitive impairment, and the mutation of *PCDH19* will affect ASD (Redies et al., 2012). And more relevant miRNAs in ASD were reviewed in Meek et al. (2013), Geaghan and Cairns (2015), and Wu et al. (2020).

MiRNAs are edited in multiple ways during their biogenesis processes (Bass et al., 1997; Luciano et al., 2004; Blow et al., 2006; Landgraf et al., 2007; Kawahara et al., 2008; Burroughs et al., 2010; de Hoon et al., 2010; Guo et al., 2011; Mizuguchi et al., 2011; Wyman et al., 2011; Alon et al., 2012; Ekdahl et al., 2012; Heo et al., 2012). A-to-I is a type of editing that has been studied in depth (Kawahara et al., 2007). The editing of A-to-I is catalyzed by the ADAR enzymes. There are three ADARs in human genome, but only ADAR1 and ADAR2 have catalytic capability to modify specific adenosines in double-stranded RNAs to inosines, which are regarded as guanines by transcriptional complexes (Wang et al., 2013). Despite its high sequence similarity to ADAR1 and ADAR2, ADAR3 has not been shown to have deaminase activity *in vitro* and has no known *in vivo* target (Chen et al., 2000). APOBEC enzymes perform the editing of C-to-U in RNAs (Negi et al., 2015; Ichinose and Sugita, 2017; Gagnidze et al., 2018). C-to-U editing sites were reported in some animal miRNAs (Zheng et al., 2014, 2016; Negi et al., 2015; Wang et al., 2019). Adding nucleotides at the 3′ end of a mature miRNA is another type of editing (Morin et al., 2008). 3′-U and 3′-A are the most common 3′ editing types. In general, 3′-U and 3′-A induce and inhibit miRNA degradation, respectively (Kim et al., 2010). The loading of mature miRNAs into RNA-induced silencing complexes (RISC) may be affected by 3′-A (Burroughs et al., 2010).

Editing could affect the functions of miRNAs in several ways. First, the nucleotides of mature miRNAs may be altered which often changes the target sets of miRNAs (Kawahara et al., 2007). Second, the secondary structure of pre-miRNAs may also be changed by editing, which could change stability and processing of miRNAs (Yang et al., 2006; Vesely et al., 2012). Similar to editing sites, SNPs affect the functions of miRNAs by regulating the transcription, processing, maturation of miRNAs or miRNA-mRNA interactions (Calin et al., 2005; Han and Zheng, 2013). SNPs and abnormal editing events are associated with serious diseases, such as chronic lymphocytic leukemia (Calin et al., 2005), glioblastoma (Choudhury et al., 2012), and melanoma (Shoshan et al., 2015).

Although A-to-I editing is widespread in brain (Eisenberg et al., 2005; Paz-Yaacov et al., 2010; Li and Church, 2013; Zaidan et al., 2018) and the editing level is gradually increasing during development (Ekdahl et al., 2012; Zaidan et al., 2018), miRNA editing in ASD is largely unknown. To comprehensively identify miRNA editing sites in ASD, we analyzed 131 miRNA-seq profiles from the brain samples of ASD patients and normal controls. We identified 70 sites with significantly different editing levels in superior frontal gyrus of ASD patients (ASD-SFG) when compared to the samples of normal controls. The editing levels of 13 A-to-I editing sites were significantly correlated with the age of normal controls, however, these significant correlations were disrupted in ASD patients, suggesting the miRNAs editing patterns were corrupted by the disease. Since ASD occurred in early childhood, we compared the ASD-SFG samples under 10 years to normal controls of the same ages and found that 117 miRNA editing sites had significantly different editing levels, indicating that ASD patients under 10 years had more severe changes in their miRNA editing patterns compared to normal controls of the same ages. One of the A-to-I editing sites, hsa-mir-376a-1_9_A_g, has a significantly higher editing level in the ASD-SFG samples than that in normal controls. We experimentally validate that the A-to-I edited miR-376a-5p directly represses *GPR85* and *NAPB*, which potentially contributes to the abnormal neurodevelopment of ASD patients. These results indicate that the editing of miRNAs in ASD is severely disturbed and provide novel insights into the etiology of ASD.

## 2. Materials and methods

### 2.1. The small RNA sequencing profiles used

As summarized in Supplementary Table S1.1, we selected 131 sRNA-seq data from different brain regions from the NCBI SRA database. These 131 miRNA-seq profiles included 20 samples from the superior frontal gyrus of the ASD patients (ASD-SFG), and 111 normal control samples with 25 superior frontal gyrus (SFG), 14 amygdala (Am), 6 Frontal Cortex (FC), 6 Corpus Callosum (CC), 3 astrocytes (As), 2 Inferior parietal lob (IPL), 2 temporal neocortex gray matter (NG), 36 prefrontal cortex (PC) and 17 unknown brain regions.

### 2.2. Genome and annotation of miRNAs used

The human unmasked genomic sequence (hg38) were downloaded from the UCSC Genome Browser (Rosenbloom et al., 2015). The index file of the human genome was generated by the bowtie-build program in the Bowtie package (Langmead et al., 2009). The pre-miRNA sequences and genomic positions of miRNAs in gff3 format were downloaded from the miRBase (release 21) (Kozomara and Griffiths-Jones, 2014).

### 2.3. Identifying mutation and editing sites in miRNAs

To identify mutation and editing (M/E) sites of pre-miRNAs, we analyzed the 131 miRNA-seq profiles selected with the MiRME pipeline (Zheng et al., 2016). Briefly, we filtered low-quality reads, then removed the 3′-adapters of raw reads. Next, we kept the unique reads, and calculated the counts of the unique reads of at least 18 nt. Then, we used NCBI BLASTN to align the unique sequence to the pre-miRNAs and obtained the unique sequences mapped to the pre-miRNAs and these unique sequences were aligned to the genome with Bowtie (v1.3.1) (Langmead et al., 2009). The cross-mapping correction method (de Hoon et al., 2010) was then used to correct the alignments to the genome. In the main step, the editing and mutation sites in the pre-miRNAs were identified by the MiRME algorithm (v1.3) using its default parameters (Zheng et al., 2016). Then, the obtained results of different samples were combined by a separate program in the MiRME package (see details in Zheng et al., 2016; Zheng, 2018).

The M/E site identified was named with the name of the pre-miRNA, M/E position in pre-miRNA, original nucleotide in upper case and the edited/mutated nucleotide in lower case. For example, hsa-mir-376a-1_9_A_g means an A-to-I editing site at the 9th nucleotide of hsa-mir-376a-1. And edited miRNA was named by the miRNA name, the M/E position in pre-miRNA, and edited/mutated nucleotide in lower case. For example, hsa-mir-376a-1_9g is the edited miR-397a-1-5p.

The criteria used to define significant M/E sites include: (i) the editing level is at least 5%; (ii) at least 10 reads support the editing event; (iii) the score threshold of sequencing reads is 30; (iv) a multiple-test corrected *P*-value (using the Benjamini and Hochberg method Benjamini and Hochberg, 1995) of smaller than 0.05. To remove M/E sites due to random sequencing errors, 834 M/E sites that had significant editing level at least 10% (13 samples) of the 131 samples used in this study were kept in further analysis.

### 2.4. Identifying conserved editing sites in miRNAs

The A-to-I and C-to-U editing sites were compared to their counterparts in *Macaca* *mulatta* (Wang et al., 2019) and *Mus* *musculus* (Kawahara et al., 2007, 2008). The editing sites of the same editing types that located on the same positions of mature miRNAs of at least two different species were considered as conserved editing sites.

### 2.5. Comparing the M/E sites to reported SNPs

The human dbSNP database (v151) was compared to the identified 834 M/E sites. The M/E sites should meet the following standard conditions, to be considered as SNP (i) have the same genomic positions as the SNP; (ii) they share the same nucleotides as SNP alleles, including primitive nucleotides and altered nucleotides; and (iii) the editing level of the M/Esite is 100% in at least one of the 131 samples.

### 2.6. Identifying age-related miRNA editing sites

We used the corr function in MATLAB (R2014b, Mathworks, MA) to calculate the Spearman correlation between the editing level of each of the 834 editing sites in Supplementary Table S2.1 and the age of death (y), as well as its *P*-value, for the 20 ASD-SFG and 25 SFG samples, respectively. The *P*-values were then corrected with the Benjamini and Hochberg method (Benjamini and Hochberg, 1995). The editing sites with corrected *P*-values smaller than 0.05 were regarded as age-related.

### 2.7. Identifying miRNA editing sites with significantly different editing levels in ASD

We examined the difference between the editing levels of 834 editing sites of 20 ASD-SFG and 25 normal SFG samples with the Mann-Whitney *U*-tests. The M/E sites with *P*-values smaller than 0.05 were considered to have significantly different editing levels in ASD.

### 2.8. Identifying miRNA editing sites with significantly different editing levels in ASD under 10 year old

We screened 9 ASD-SFG and 15 normal SFG samples under the age of 10 (y) from the entire sample. Then we examined the difference between the editing levels of 834 editing sites of 9 ASD-SFG and 15 normal SFG samples with the Mann-Whitney *U*-tests. The obtained *P*-value were also corrected with the Benjamini-Hochberg correction method (Benjamini and Hochberg, 1995). The M/E sites with *P*-values smaller than 0.05 were considered to have significantly different editing levels in ASD under 10 year old.

### 2.9. Analyzing putative targets of edited miRNAs in ASD

One editing site, hsa-mir-376a-1_9_A_g, occurring in seed region of mature miRNA was selected from 70 significant editing sites. Then, the target genes of edited hsa-miR-376a-5p, i.e., hsa-mir-376a-1_9g, were identified with the MiCPAR algorithm (Zheng, 2018). We combined 11 PAR-CLIP sequencing profiles as listed in Supplementary Table S1.2 and the combined profile was used to identify targets of original and A-to-I edited hsa-miR-376a-5p. Because the editing level of hsa-mir-376a-1_9_A_g was significantly increased in the ASD-SFG samples when compared with normal SFG samples, the identified targets of edited hsa-miR-376a-1-5p with at least one PAR-CLIP read and the down-regulated genes in ASD gene expression profiles were compared to find common genes.

### 2.10. Gene expression data sets of brain samples of ASD patients

In order to understand the potential functions of the A-to-I edited hsa-miR-376a-5p, we examined the dysregulated genes in the brain samples from ASD patients and normal controls. Since the editing level of hsa-mir-376a-1_9_A_g was significantly up-regulated in the ASD-SFG samples when compared with normal SFG samples, we chose the target genes that were commonly down-regulated in three cohorts of gene expression profiles of ASD patients in literature (Irimia et al., 2014; D'Gama et al., 2015; Parikshak et al., 2016). The expression data in Irimia et al. (2014) included 12 ASD superior temporal gyrus (ASD-STG) samples and 12 normal control superior temporal gyrus (STG) samples. We identified 1,499 significantly down-regulated genes (*P* < 0.05, edgeR Robinson et al., 2010) in ASD-STG samples. Data sets reported in Parikshak et al. includes 85 ASD frontal (ASD-FC1) and temporal cortex (ASD-TC1) samples and 82 normal control frontal (FC1) and temporal cortex (TC1) samples (Parikshak et al., 2016). Previous studies identified 558 significantly down-regulated genes (multiple test corrected *P* < 0.05, linear mixed effect (LME) model) in ASD-FC1 and ASD-TC1 samples (Parikshak et al., 2016). Data sets reported by D'Gama et al. (2015) included 6 ASD prefrontal cortex (ASD-PFC1) samples and 12 normal control prefrontal cortex (PFC1) sample. We identified 400 significantly down-regulated genes (*P* < 0.05, limma Ritchie et al., 2015) in ASD-PFC1 samples.

As summarized in Supplementary Table S1.3, we selected 9 cohorts of gene expression profiles of ASD brain samples to examine the expression of ADARs. These included dorsolateral prefrontal cortex (DLPFC) (GSE102741), superior temporal gyrus (STG) (GSE64018), brain tissue (BT) (GSE28475), cerebellum (CE1) (GSE28521), frontal cortex (FC2) (GSE28521), temporal cortex (TC2) (GSE28521), cerebellum (CE2) (GSE38322), occipital cortex (OC-BA19) (GSE38322), and corpus callosum (CC) (GSE62098). The dysregulated genes in seven ASD gene expression data sets, i.e., DLPFC, BT, CE1, FC2, TC2, CE2, and OC-BA19 were identified through the limma package (Ritchie et al., 2015). The differentially expressed genes in the STG and CC were identified with the edgeR package (Robinson et al., 2010).

### 2.11. Plasmid construction

We synthesized about 180 base pairs in the genomic region of hsa-mir-376a-1 and another sequence with hsa-mir-376a-1_9_A_g, and cloned them into the plasmid pCDNA 3.1(+) (Invirtrogen, Carlsbad, CA, USA) with BamHl and EcoRl sites, which were named as p376a-1 and p376a-1_9g, respectively. The regions with putative hsa-mir-376a-1_9g binding sites of in the 3′ UTRs of *GPR85* and *NAPB* (with 20 nt on both sites of the miR-376a-1_9g complementary sites) were cloned into the pmirGLO vectors (Promega, Madison, WI, US) with Nhel and Xhol sites, named as pGLO-GPR85 and pGLO-NAPB, respectively. The pmirGLO vector itself provided Renilla luciferase signal as a control. The 3′-UTR regions of *GPR85* and *NAPB* with mutated hsa-mir-376a-1_9g binding sites were also cloned into pmiRGLO vectors, named as pGLO-GPR85m and pGLO-NAPBm, respectively.

### 2.12. Luciferase reporter assay

Human renal epithelium cell lines (293T) were purchased from Cell Bank of the Kunming Institute of Zoology, Chinese Academy of Sciences (Kunming, China). Human 293T cells were grown in DMEM containing 10% FBS and co-transfected with one of the p376a-1, p376a-1_9g, and pCDNA3.1 empty vector at a final concentration of 500 ng/μl, and one of pGLO-GPR85, pGLO-GPR85m, pGLO-NAPB, pGLO-hsa-NAPBm, and pGLO empty vector with the final transfection concentration of 500 ng/μl. Lipofectamine 2000 (Invitrogen, Carlsbad, CA, USA) was used and transfection was performed on a 24-well plate according to the instructions. TransDetect double-luciferase Reporter Assay Kit (Beijing TransGen Biotech, Beijing, China) was used to detect luciferase activities for each of the biological replicates with three technical repeats. The average of the three biological replicates were taken as the final fluorescence result. The luciferase activities of different groups were then compared with two tailed *t*-tests.

### 2.13. Statistics and reproducibility

Spearman correlation was used to examine the correlation between editing levels of the 834 editing sites and the ages (at death) of 25 SFG and 20 ASD-SFG samples, respectively. Mann-Whitney *U*-test was used to examine M/E sites for different editing levels between ASD-SFG samples and SFG samples. EdgeR (Robinson et al., 2010) implemented in R (v4.1), limma (Ritchie et al., 2015) implemented in R (v4.1) and two-tailed *t*-tests were used to compare gene expression levels in ASD and control samples, including *ADAR1, ADAR2, ADAR3*,*GPR85, NAPB*, and *PRPS1*. Spearman correlation, Mann-Whitney *U*-tests and two-tailed *t*-tests were performed using MATLAB (R2014b, MathWorks, MA). All specific *P*-values and sample numbers were listed in Supplementary material.

## 3. Results

### 3.1. Summary of miRNA mutation and editing sites identified

**Figure 1:**
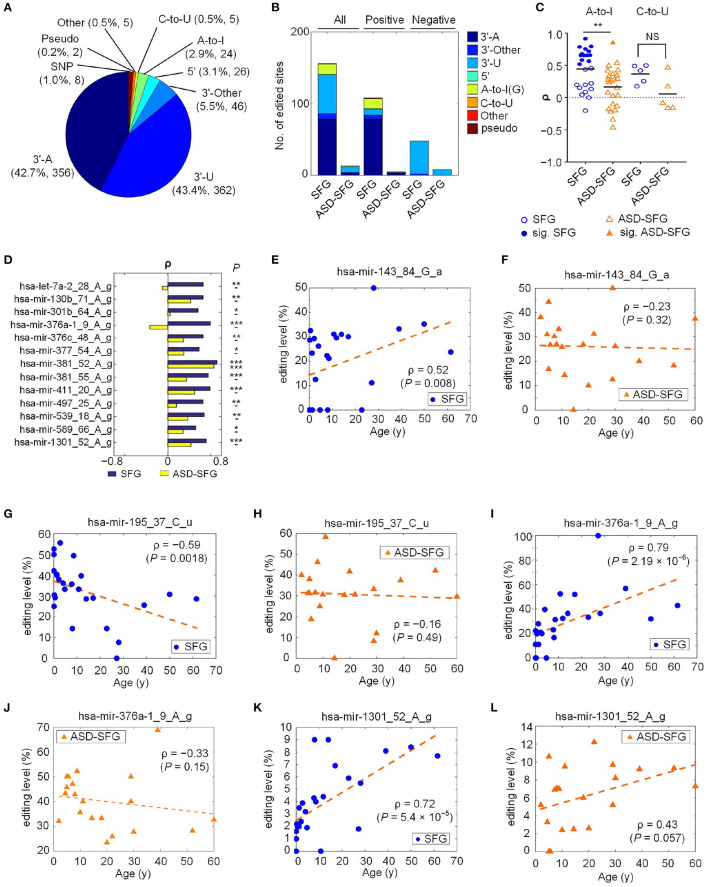
A summary of the identified miRNA M/E sites in ASD. The detailed legend is provided on the next page. A summary of the identified miRNA M/E sites in ASD. **(A)** The categories of significant M/E sites in miRNAs. **(B)** The numbers of different types of editing sites that have significant Spearman correlation, ρ, between editing levels and the ages of individuals in the SFG and ASD-SFG group. **(C)** The distributions of the ρ values of the A-to-I and C-to-U sites in the SFG and ASD-SFG samples, respectively. Solid marks indicate those sites with significant ρ values. **: *P* < 0.01; and NS: not significant. **(D)** Thirteen selected editing sites with significant ρ values in either SFG or ASD-SFG samples. *P < 0.05, **P < 0.01, and ***P < 0.001. **(E)** ρ between the ages of SFG samples and the editing level of hsa-mir-143_84_G_a. **(F)** ρ between the ages of ASD-SFG samples and the editing level of hsa-mir-143_84_G_a. **(G)** ρ between the ages of SFG samples and the editing level of hsa-mir-195_37_C_u. **(H)** ρ between the ages of ASD-SFG samples and the editing level of hsa-mir-195_37_C_u. **(I)** ρ between the ages of SFG samples and the editing level of hsa-mir-376a-1_9_A_g. **(J)** ρ between the ages of ASD-SFG samples and the editing level of hsa-mir-376a-1_9_A_g. **(K)** ρ between the ages of SFG samples and the editing level of hsa-mir-1301_52_A_g. **(L)** ρ between the ages of ASD-SFG samples and the editing level of hsa-mir-1301_52_A_g.

To identify mutation and editing (M/E) events of miRNAs in ASD, we collected 20 miRNA-seq profiles of superior frontal gyrus samples of the ASD patients (ASD-SFG) and 111 miRNA-seq profiles of different regions of normal controls from the NCBI SRA database (as listed in Supplementary Table S1). These miRNA-seq profiles were analyzed with the MiRME algorithm (Zheng et al., 2016) to identify M/E sites in miRNAs. We identified a total of 834 significant M/E sites which had at least 5% editing levels and were supported by at least 10 normalized sequencing reads (Reads Per Ten Million sequencing tags) in at least 10% (13) of the selected samples (as listed in Supplementary Table S2.1). As shown in Figure 1A and Supplementary Table S6, based on the positions of the editing sites, nucleotide variation in editing events, and SNP annotation (see Zheng et al., 2016), these 834 M/E sites are classified into nine different categories: 3′-A, 3′-U, 3′-Other, 5′, A-to-I, C-to-U, Other, Pseudo, and SNP. Among the 834 editing sites, the first two largest categories are 3′-U and 3′-A, accounting for 43.4 and 42.7%, respectively.

Next, we examined several different types of editing sites that did not occur at both ends of the mature miRNAs. Totally, there were 24 A-to-I, 5 C-to-U, and 3 U-to-G editing sites (Supplementary Figure S1A and Supplementary Table S12). Similar to previous work (Zheng et al., 2016), there may be several 3′-editing sites in one pre-miRNA, but most pre-miRNAs only have 1 or 2 central sites in our selected samples (Supplementary Figure S1B). However, some miRNAs may have a few editing events at 5′ ends similar to our previous studies (Zheng et al., 2016; Wang et al., 2019; Guo et al., 2022).

### 3.2. Age-related editing sites in ASD and normal controls

Because previous studies reported that the editing levels of A-to-I editing sites in miRNAs were gradually increasing in the developmental process (Ekdahl et al., 2012; Zaidan et al., 2018), we examined the Spearman correlation (ρ) between editing levels of the 834 editing sites and the ages (at death) of 25 SFG and 20 ASD-SFG samples, respectively. There are 155 sites with significant ρ values in SFG, but only 13 sites with significant ρ values in ASD-SFG samples (Figure 1B and Supplementary Tables S2.2, 2.3). The numbers of significant editing sites with positive or negative correlations in SFG samples are much larger than those in ASD-SFG samples (Figure 1B and Supplementary Figure S1C). The numbers of types of editing sites are also decreased in the ASD-SFG samples. The numbers of types of editing sites are 6 in the SFG samples, and the numbers of types of editing sites are 3 in the ASD-SFG samples. These results suggest that the gradually increasing or decreasing editing levels of miRNAs are severely disturbed in ASD.

We carefully examined the values of 24 A-to-I and 5 C-to-U editing sites (Figure 1C and Supplementary Table S6). In SFG samples, the mean ρ value of A-to-I sites is 0.46 (Figure 1C), which is consistent with previous results that A-to-I editing levels increase with age (Ekdahl et al., 2012; Zaidan et al., 2018). In ASD-SFG samples, the mean ρ value of A-to-I sites decreases severely to 0.15 (Figure 1C), indicating that the A-to-I editing of miRNAs is disrupted in ASD. Similarly, in normal controls, the positive mean ρ value of the C-to-U editing sites is 0.37, suggesting that the editing level of C-to-U sites usually increases when people are aging. In ASD-SFG samples, the mean ρ value of C-to-U sites decreases to 0.06, indicating that C-to-U editing of miRNAs is also dysregulated in ASD. In SFG samples, there are 13 A-to-I sites with significant positive values (Figure 1D). In comparison, only 1 A-to-I site has significant positive values in ASD-SFG samples (Figure 1D).

In normal controls, the editing levels of many A-to-I and 3′-A sites were positively correlated with ages, however the editing levels of most 3′-U sites were negatively correlated with ages (Figure 1B and Supplementary Table S6). As shown in Figures 1E, G, the editing level of a 3′-A site, hsa-mir-143_84_G_a, was positively correlated with age and the editing level of a 3′-U site, hsa-mir-195_37_C_u, was negatively correlated with age, respectively. But these two sites did not show significant correlation with ages in ASD-SFG samples (Figures 1F, H, respectively).

As shown in Figures 1I, K, the editing levels of hsa-mir-376a-1_9_A_g and hsa-mir-1301_52_A_g had significant correlations with ages in normal controls, but these correlations were interrupted (Figure 1L) or even reversed (Figure 1J) in ASD patients.

### 3.3. A-to-I editing sites

As shown in Supplementary Figure S2A and Supplementary Table S2.4, we detected 24 A-to-I editing sites with significant editing levels. As shown in Supplementary Figure S2A, hsa-mir-376a-2_55_A_g and hsa-mir-376c_48_A_g are widely reported (Kawahara et al., 2007; Zheng et al., 2016) and show high editing levels in superior frontal gyrus (SFG), amygdala (Am), frontal cortex (FC), inferior parietal lob (IPL), neocortex gray (NG), prefrontal cortex (PC) and other brain samples.

As shown in Supplementary Figure S2B and Supplementary Table S13, similar to previous results (Kawahara et al., 2008; Alon et al., 2012; Zheng et al., 2016; Wang et al., 2019; Guo et al., 2022), we can see that the 5′ and 3′ nucleotides beside the 24 A-to-I editing sites have strong preferences of being U and G, respectively.

For examples, the details for the two A-to-I are shown in Supplementary Figures S2C–F. As reported in the literature (Kawahara et al., 2007; Zheng et al., 2016), hsa-mir-376c_48_A_g is a conservative editing sites and has a high editing level 89.9% in one of the normal inferior parietal lob (IPL) samples (Supplementary Figure S2C and Supplementary Table S13). And hsa-mir-411_10_A_g is also wildly reported and has an editing level of 23.1% in one of the normal amygdala samples (Supplementary Figure S2D and Supplementary Table S13). The reads that support these sites are shown in Supplementary Figures S2E, F, respectively.

### 3.4. C-to-U editing sites

As shown in Supplementary Figure S3A and Supplementary Table S2.5, we identified 5 C-to-U editing sites, but their editing levels were relatively low in the 131 samples. Three of these 5 C-to-U editing sites are conserved in primates (Wang et al., 2019) (Supplementary Figure S3A). The neighboring nucleotides of these 5 C-to-U editing sites prefer to be C on both the 5′ and 3′ sides (Supplementary Figure S3B and Supplementary Table S14), which is consistent with the CCC motif of APOBEC3G (Chen and MacCarthy, 2017). Details of the two C-to-U editing sites are shown in Supplementary Figures S3C, D and the reads supporting these sites are shown in Supplementary Figures S3E, F.

### 3.5. Identified SNPs in miRNAs of ASD

By comparing the M/E sites to SNPs reported in dbSNP and examining their editing levels, we identified 8 SNPs in 834 M/E sites (Supplementary Figure S4A and Supplementary Table S2.6). Two of these 8 SNPs in miRNAs were shown in Supplementary Figures S4B–E and Supplementary Table S15. As shown in Supplementary Figures S4D, E, all the sequencing reads carried the variations for the two SNPs in the two samples, suggesting that these variations happened at DNA levels.

### 3.6. Relevant miRNA editing sites in ASD

**Figure 2:**
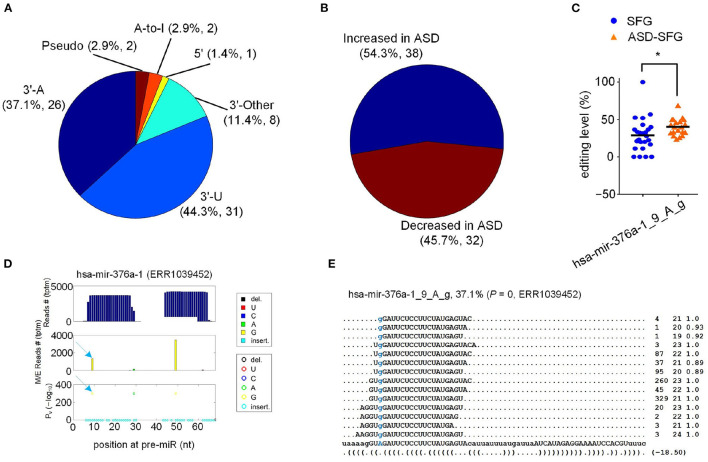
70 M/E sites that have significantly different editing levels in ASD-SFG when compared to SFG samples. **(A)** The categories of the 70 M/E sites. **(B)** The percentages of M/E sites with increased and decreased editing levels in ASD. **(C)** Comparison of the editing level of hsa-mir-376a-1_9_A_g in the ASD-SFG and SFG samples. *P < 0.05, Mann-Whitney *U*-test. **(D)** The MiRME map of hsa-mir-376a-1_9_A_g in one of the unknown brain region samples (ERR1039452). **(E)** The details of hsa-mir-376a-1_9_A_g in ERR1039452.

We then compared the editing levels of the 834 editing sites in the 20 ASD-SFG to those of the 25 SFG samples of normal people. As shown in Figure 2A and Supplementary Table S3, we identified 70 M/E sites (*P* < 0.05, Mann-Whitney *U*-tests) with significantly different editing levels in ASD-SFG samples when compared to SFG samples. The largest categories of these M/E sites were 3′-U and 3′-A, accounting for 44.3 and 37.1%, respectively. As shown in Figure 2B and Supplementary Table S7, 38 M/E sites were significantly hyperedited in ASD-SFG samples, and 32 M/E sites were significantly hypoedited in ASD-SFG samples. As shown in Figure 2C and Supplementary Table S7, hsa-mir-376a-1_9_A_g showed significantly higher editing levels in the ASD-SFG group (*P*= 0.01, Mann-Whitney *U*-test), and the reads that supported hsa-mir-376a-1_9_A_g in one of the selected samples were shown in Figures 2D, E.

### 3.7. Exploring miRNA editing in children ASD patients

ASD is an early neurodevelopmental disorder that usually develops before the age of 2–4 years (Geschwind, 2009; Barger et al., 2013; Baio et al., 2018). In infants and children, ASD could manifest itself within the first 2 years of age as delays or deficits in joint attention, imitation, and communication (Lai et al., 2013). The data obtained from 11 ADDM network sites in the United States in 2010 (Baio et al., 2018) indicates that the prevalence of ASD at 8 year old is 14.7%, suggesting that the impact of ASD in early childhood is relatively large.

**Figure 3:**
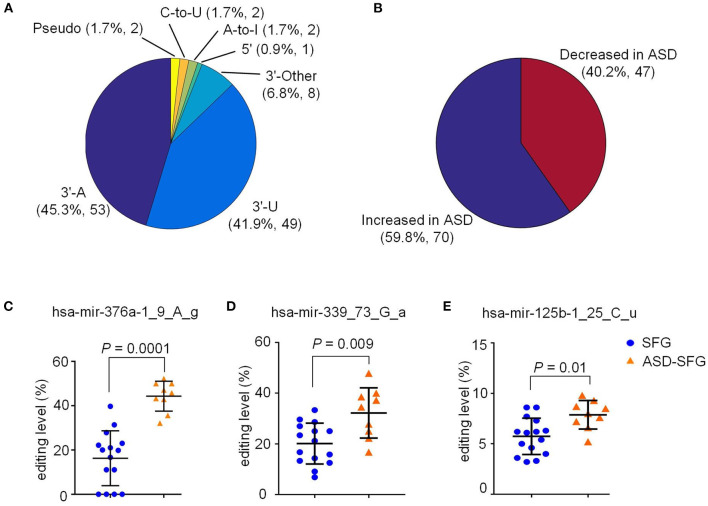
Distribution of 117 M/E sites that have significantly different editing levels in ASD samples under 10 year old when compared to controls of the same ages. **(A)** The distribution of the different types of editing sites that have significantly different editing levels in ASD-SFG samples compared to the SFG samples. **(B)** The distribution of sites in Part (a) whose editing levels are increased or decreased in ASD-SFG samples when compared to SFG samples. **(C)** Comparison of the editing level of hsa-mir-376a-1_9_A_g in ASD-SFG and SFG samples under 10 year old. **(D)** Comparison of the editing level of hsa-mir-339_73_G_a in ASD-SFG and SFG samples under 10 year old. **(E)** Comparison of the editing level of hsa-mir-125b-1_25_C_u in ASD-SFG and SFG samples under 10 year old. NS, not significant.

To further explore the change of miRNA editing in children ASD patients, we compared miRNA editing sites of ASD-SFG brain samples before the age of 10 (y) to normal controls of the same ages. As shown in Figure 3A and Supplementary Table S4, we identified 117 M/E sites with significantly different editing levels (*P* < 0.05, Mann-Whitney *U*-tests) in ASD-SFG samples when compared to SFG controls. The largest categories of these M/E sites were also 3′-A and 3′-U, which was 45.3 and 41.9% respectively. As shown in Figure 3B and Supplementary Table S8, 70 M/E sites were significantly hyperedited and 47 M/E sites were significantly hypoedited in ASD-SFG samples under 10 year old when compared to SFG samples of the same ages.

As shown in Figure 3C and Supplementary Table S8, the editing level of hsa-mir-376a-1_9_A_g is very high in ASD-SFG under 10 years, and relatively low in SFG of normal controls under 10 years. As expected, the editing level of hsa-mir-376a-1_9_A_g shows more severe increases in ASD-SFG under 10 years (*P*= 0.0001, Mann-Whitney *U*-test) than SFG normal controls under 10 years (Figure 3C), in comparison to results of all samples in Figure 2C.

As shown in Figures 3D, E, the editing levels of hsa-mir-339_73_G_a, and hsa-mir-125b-1_25_C_u in ASD-SFG samples under 10 year old were significantly higher than control samples.

### 3.8. The expression patterns of ADARs in ASD

**Figure 4:**
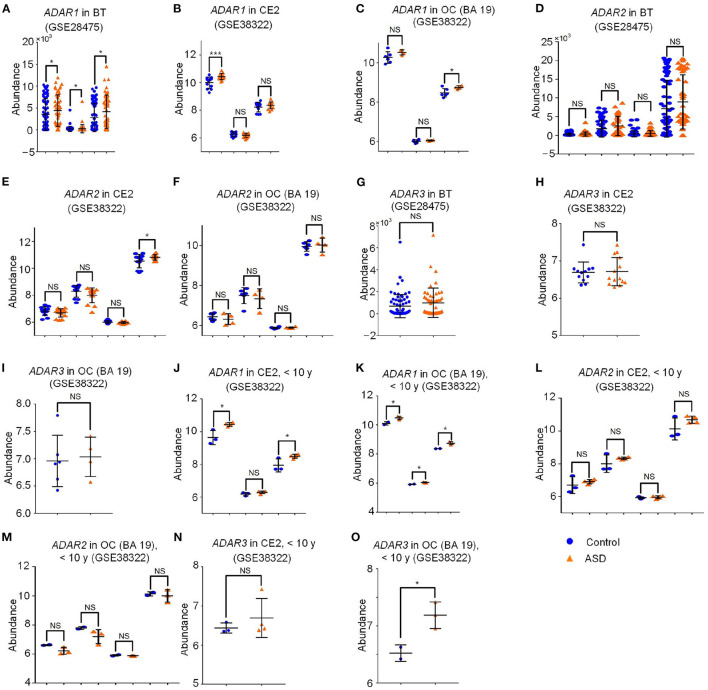
The abundance of ADARs in different brain regions of ASD patients and normal controls. The detailed legend is provided on the next page. The abundance of ADARs in different brain regions of ASD patients and normal controls. **(A)** The abundance of ADAR1 (ILMN_1776777, ILMN_1706963 and ILMN_2320964) in postmortem brain samples (BT) of ASD patients and normal controls (GSE28475). **(B)** The abundance of ADAR1 (ILMN_1776777, ILMN_1706963 and ILMN_2320964) in cerebellum (CE2) samples of Normal controls (NC) and ASD patients (ASD) (GSE38322). **(C)** The abundance of ADAR1 (ILMN_1776777, ILMN_1706963 and ILMN_2320964) in occipital cortex (OC) (BA 19) samples of Normal controls (NC) and ASD patients (ASD) (GSE38322). **(D)** The abundance of ADAR2 (ILMN_1697628, ILMN_2319326, ILMN_1657442 and ILMN_1679797) in postmortem brain samples (BT) of ASD patients and normal controls (GSE28475). **(E)** The abundance of ADAR2 (ILMN_1697628, ILMN_2319326, ILMN_1657442 and ILMN_1679797) in cerebellum (CE2) samples of Normal controls (NC) and ASD patients (ASD) (GSE38322). **(F)** The abundance of ADAR2 (ILMN_1697628, ILMN_2319326, ILMN_1657442 and ILMN_1679797) in occipital cortex (OC) (BA 19) samples of Normal controls (NC) and ASD patients (ASD) (GSE38322). **(G)** The abundance of ADAR3 (ILMN_1749493) in postmortem brain samples (BT) of ASD patients and normal controls (GSE28475). **(H)** The abundance of ADAR3 (ILMN_1749493) in cerebellum (CE2) samples of Normal controls (NC) and ASD patients (ASD) (GSE38322). **(I)** The abundance of ADAR3 (ILMN_1749493) in occipital cortex (OC) (BA 19) samples of Normal controls (NC) and ASD patients (ASD) (GSE38322). **(J)** The abundance of ADAR1 (ILMN_1776777, ILMN_1706963 and ILMN_2320964) in cerebellum (CE2) samples of Normal controls (NC) and ASD patients (ASD) under 10 year old (GSE38322). **(K)** The abundance of ADAR1 (ILMN_1776777, ILMN_1706963 and ILMN_2320964) in occipital cortex (OC) (BA 19) samples of Normal controls (NC) and ASD patients (ASD) under 10 year old (GSE38322). **(L)** The abundance of ADAR2 (ILMN_1697628, ILMN_2319326, ILMN_1657442 and ILMN_1679797) in cerebellum (CE2) samples of Normal controls (NC) and ASD patients (ASD) under 10 year old (GSE38322). **(M)** The abundance of ADAR2 (ILMN_1697628, ILMN_2319326, ILMN_1657442 and ILMN_1679797) in occipital cortex (OC) (BA 19) samples of Normal controls (NC) and ASD patients (ASD) under 10 year old (GSE38322). **(N)** The abundance of ADAR3 (ILMN_1749493) in cerebellum (CE2) samples of Normal controls (NC) and ASD patients (ASD) under 10 year old (GSE38322). **(O)** The abundance of ADAR3 (ILMN_1749493) in occipital cortex (OC) (BA 19) samples of Normal controls (NC) and ASD patients (ASD) under 10 year old (GSE38322). In Part **(A–I)**, the *P*-values were calculated with the limma package (Ritchie et al., 2015). In Part **(J–O)**, the *P*-values were calculated with the two-tailed *t*-tests. *P < 0.05, ***P < 0.001, and NS, not significant.

A-to-I editing of RNA is catalyzed by the ADAR1 and ADAR2 (also known as ADARB1) (Wang et al., 2013), and is repressed by ADAR3 (i.e., ADARB2) (Kurup et al., 2022). We thus examined the expression levels of *ADAR1, ADAR2*, and *ADAR3* in different brain regions of ASD patients. The expression levels of *ADAR1* (ILMN_1776777, ILMN_1706963, and ILMN_2320964) were significantly up-regulated in brains of postmortem ASD patients (GSE28475) compared to normal controls (Figure 4A and Supplementary Table S9), the expression levels of *ADAR2* (ILMN_1697628, ILMN_2319326, ILMN_1657442, and ILMN_1679797) and *ADAR3* (ILMN_1749493) showed non-significant increasing trends in brains (BT) of postmortem ASD patients (GSE28475) (Figures 4D, G). The expression levels of *ADAR1* (ILMN_1776777) and *ADAR2* (ILMN_1679797) in cerebellum (CE2) of ASD patients (GSE38322) were significantly up-regulated compared to the normal controls. However, different probes of *ADAR1* and *ADAR2*, and *ADAR3* (ILMN_1749493) showed non-significant changes in cerebellum (CE2) of ASD patients (GSE38322) (Figures 4B, E, H). The expression levels of *ADAR1* (ILMN_2320964) in occipital cortex (OC) (BA19) of ASD patients (GSE38322) were significantly up-regulated compared to the normal controls. Similarly, different probes of *ADAR1* (ILMN_1776777 and ILMN_1706963), ADAR2 (ILMN_1697628, ILMN_2319326, ILMN_1657442, and ILMN_1679797), and ADAR3 (ILMN_1749493) show non-significant variations in OC (BA19) of ASD patients (GSE38322) (Figures 4C, F, I). The expression levels of *ADAR1, ADAR2*, and *ADAR3* showed non-significant changes in superior temporal gyrus (STG) (GSE64018), corpus callosum (CC) (GSE62098), cerebellum (CE1), frontal cortex (FC2), temporal cortex (TC2) (GSE28521), dorsolateral prefrontal cortex (DLPFC) (GSE102741) (Supplementary Figures S5A–F and Supplementary Table S16). In summary, the increased expression of *ADAR1* is consistent with the increased editing levels of the two A-to-I editing sites hsa-mir-376a-1_9_A_g and hsa-mir-1301_52_A_g (Figures 2A, C and Supplementary Table S3) in ASD-SFG samples compared to SFG samples. However, more studies dedicated to different brain regions are necessary to further validate the relation between ADARs and editing levels of hsa-mir-376a-1_9_A_g and hsa-mir-1301_52_A_g. Furthermore, our results also suggest that possible therapies of ASD patients could be designed by reducing the expression of *ADAR1*.

Next we examined the expression of ADARs in samples under 10 year old. As shown in Figures 4J, K, the expression level of *ADAR1* was significantly up-regulated in CE2 (ILMN_1776777 and ILMN_2320964) and OC (BA19) samples (ILMN_1776777, ILMN_1706963 and ILMN_2320964) of ASD patients under 10 year old (GSE38322). In addition, the expression level of *ADAR3* (ILMN_1749493) was significantly up-regulated in the ASD brain samples before the age of 10 in OC (BA19) samples (GSE38322) (Figure 4O) and did not change significantly in CE2 of ASD patients under 10 years (Figure 4N). *ADAR2* (ILMN_1697628, ILMN_2319326, ILMN_1657442 and ILMN_1679797) did not show severe change in CE2 and OC (BA19) of ASD patients under 10 years compared to normal controls (Figures 4L, M, respectively). The expression levels of *ADAR1, ADAR2*, and *ADAR3* in dorsolateral prefrontal cortex (DLPFC) (GSE102741) showed non-significant down-regulation compared to the normal controls under 10 years (Supplementary Figure S6A and Supplementary Table S17). When comparing the results in Figures 4B–J, C–K , it could be noticed that ADAR1 showed a clearer upregulation in ASD samples under 10 years compared to normal controls. This suggests that *ADAR1* might be a key factor that contributes to the more severely increased editing levels of hsa-mir-376a-1_9_A_g and hsa-let-7a-2_28_A_g in ASD patients under 10 years (Figure 3C and Supplementary Table S4).

### 3.9. Target analysis of A-to-I editing sites in seed regions of miRNAs

**Figure 5:**
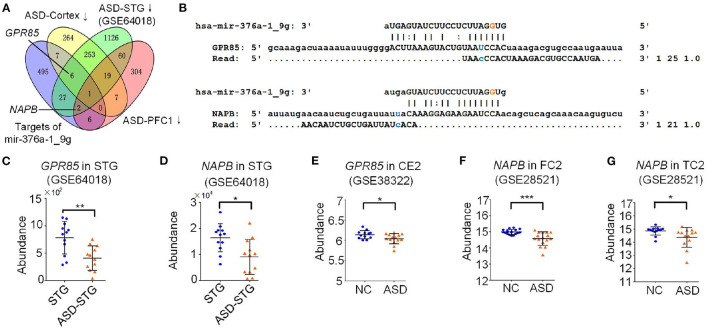
The target analysis of A-to-I edited hsa-miR-376a-5p. **(A)** Integrated analysis of targets of edited hsa-miR-376a-5p (hsa-miR-376a-1_9g) and the downregulated genes in different ASD brain regions. ASD-Cortex means the down-regulated gene when comparing ASD-FC1+ASD-TC1 to FC1+TC1. ASD-STG means the down-regulated gene when comparing ASD-STG to STG. ASD-PFC1 means the down-regulated gene when comparing ASD-PFC1 to PFC1. **(B)** The complementary sites of hsa-miR-376a-1_9g on *GPR85* and *NAPB*, and the PAR-CLIP sequencing reads from these sites. **(C)** The abundance of *GPR85* in superior temporal gyrus (STG) samples of Normal controls (NC) and ASD patients (ASD) (GSE64018). *P < 0.05, **P < 0.01, and ***P < 0.001, edgeR package (Robinson et al., 2010). **(D)** The abundance of *NAPB* in superior temporal gyrus (STG) samples of Normal controls (NC) and ASD patients (ASD) (GSE64018). *P < 0.05, **P < 0.01, and ***P < 0.001, edgeR package (Robinson et al., 2010). **(E)** The abundance of *GPR85* (ILMN_1748338) in cerebellum (CE2) samples of Normal controls (NC) and ASD patients (ASD) (GSE38322). *: *P* < 0.05; **: *P* < 0.01; ***: *P* < 0.001, limma package (Ritchie et al., 2015). **(F)** The abundance of *NAPB* (ILMN_2181125) in Frontal cortex (FC2) samples of Normal controls (NC) and ASD patients (ASD) (GSE28521). *P < 0.05, **P < 0.01, and ***P < 0.001, limma package (Ritchie et al., 2015). **(G)** The abundance of *NAPB* (ILMN_2181125) in temporal cortex (TC2) samples of normal controls (NC) and ASD patients (ASD) (GSE28521). *P < 0.05, **P < 0.01, and ***P < 0.001, limma package (Ritchie et al., 2015).

Fifteen of the 24 A-to-I editing sites located in the seed regions of mature miRNAs, suggesting that these A-to-I editing events might change the targets of the miRNAs. Only two A-to-I editing sites showed significantly different editing levels when comparing ASD-SFG to SFG samples (Supplementary Table S3). We selected one of these two sites, i.e., hsa-mir-376a-1_9_A_g, and used the MiCPAR pipeline to predict the targets of the original and edited hsa-miR-376a-1-5p (Zheng, 2018), as listed in Supplementary Table S5.1 and Supplementary Table S5.2, respectively. We next carefully examined the targets of hsa-mir-376a-1_9g. We compared the targets of hsa-mir-376a-1_9g and the down-regulated genes in brain samples of ASD patients in Figure 5A and Supplementary Table S10, because the editing level of hsa-mir-376a-1_9_A_g significantly increased in brains of ASD patients. We found that hsa-mir-376a-1_9g targeted G protein-coupled receptor 85 (*GPR85*) and NSF attachment protein beta (*NAPB*) (Figure 5B). Presumably due to increased editing level of hsa-mir-376a-1_9g in ASD-SFG (Figure 2A), the expression levels of *GPR85* and *NAPB* are significantly downregulated in superior temporal gyrus (STG), cerebellum (CE2), frontal cortex (FC2), and temporal cortex (TC2) regions of ASD patients (Figures 5C–G). However, the expression levels of *GPR85* and *NAPB* did not show universal down-regulation in different brain regions. The expression of *GPR85* and *NAPB* showed non-significant decreases in the corpus callosum (CC), and occipital cortex (OC) (BA19) (Supplementary Figures S7A, B, S8A–C and Supplementary Tables S18, S19, respectively); and showed slight increase in BT, and DLPFC (Supplementary Figures S7C, D, S8E, F and Supplementary Tables S18, S19, respectively).

Importantly, there is a significant positive correlation between the editing level of hsa-mir-376a-1_9_A_g and ages of individuals in SFG of normal people (Figure 1I). There was a nonsignificant negative Spearman correlation between expression of *GPR85* and ages in STG, CE2, and DLPFC (Supplementary Figures S7E–G, respectively), and the Spearman correlation between expression of *NAPB* and ages in STG, CE2, and DLPFC were positive and nonsignificant (Supplementary Figures S8G–I, respetively). In ASD, the correlations of *NAPB* were weakened in CE2 and DLPFC (Supplementary Figures S8K, L, respectively), but the negative correlation of *GPR85* was slightly enhanced in STG and DLPFC (Supplementary Figures S7H, I, respectively), and the correlation of *NAPB* in STG became negative (Supplementary Figure S8J).

### 3.10. hsa-mir-376a-1_9g directly represses *GPR85* and *NAPB*

**Figure 6:**
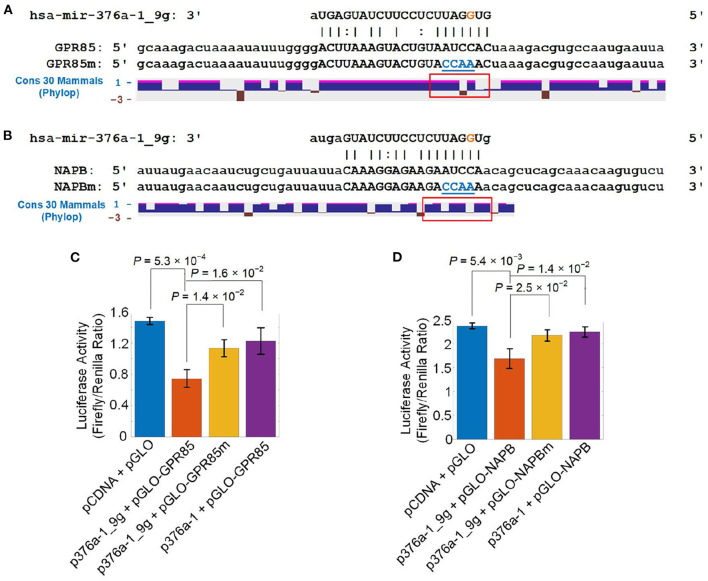
Validating that A-to-I edited miR-376a-1-5p directly represses *GPR85* and *NAPB*. **(A)** The segment of 3′ UTR of *GPR85*, mutated segment of 3′ UTR of *GPR85* and conservation scores for 30 mammals in this region calculated with PhyloP. **(B)** The segment of 3′ UTR of *NAPB*, mutated segment of 3′ UTR of *NAPB* and conservation scores for 30 mammals in this region calculated with PhyloP. **(C)** The luciferase activities when co-transfecting a pGLO plasmid of 3′ UTRs of hsa *GPR85* in Part **(A)** and a pCDNA plasmid containing original pre-hsa-mir-376a (p376a-1) or pre-hsa-mir-376a-1_9g (p376a-1_9g). **(D)** The luciferase activities when co-transfecting a pGLO plasmid of 3′ UTRs of hsa *NAPB* in Part **(B)** and p376a-1 or p376a-1_9g. In Part **(A, B)**, the regions in the red rectangles were 3′-UTR parts opposite to the seed region of hsa-mir-376a-1_9g. The blue nucleotides in GPR85m and NAPBm were the mutated nucleotides. In Part **(C, D)**, the values shown are mean ± SD. *P*-values are based on two-tailed *t*-tests.

We first examined the conservation of the complementary site of hsa-mir-376a-1_9g on *GPR85* and *NAPB*. The regions opposite to the seed of edited miR-376a-5p were only partially conserved in mammals (Figures 6A, B and Supplementary Table S11, respectively).

In order to verify that hsa-mir-376a-1_9g directly repressed *GPR85* and *NAPB*, we co-transfected a plasmid including pre-mir-376a-1 with or without the edited nucleotide and another plasmid with the original *GPR85*/*NAPB* 3′-UTR segments with the complementary sites of hsa-mir-376a-1_9g or the same segments with mutated complementary sites of hsa-mir-376a-1_9g. hsa-mir-376a-1_9g significantly reduced the luciferase activities of *GPR85* and *NAPB* (Figures 6C, D and Supplementary Table S11), indicating that hsa-mir-376a-1_9g directly repressed both *GPR85* and *NAPB*. Because *GPR85* and *NAPB* of humans and monkeys were conserved in primate (Supplementary Figures S9A, B), we performed the luciferase assays for the same *GPR85*/*NAPB* segments of monkey. hsa-mir-376a-1_9g significantly decreased the luciferase activities of monkey *GPR85* and monkey *NAPB* (Supplementary Figures S9C, D and Supplementary Table S20), indicating that hsa-mir-376a-1_9g directly repressed monkey *GPR85* and monkey *NAPB*. In comparison, when being co-transfected with mutated *GPR85* and mouse *Gpr85* (Supplementary Figure S9G), the mutated *GPR85* and mouse *Gpr85* could not be repressed by the hsa-mir-376a-1_9g (Supplementary Figure S9E and Supplementary Table S20). The mutated *GPR85* and mouse *Gpr85* could not be repressed by the original miR-376a-5p too (Supplementary Figure S9E). The complementary site of hsa-mir-376a-1_9g on mouse *Napb* only had one nucleotide different (U opposite to the third nucleotide, i.e., the A-to-I editing site, of hsa-mir-376a-1_9g) from the cytosines of human and monkey NAPB (Supplementary Figure S9H). Presumably, because a G-to-U pair formed at the different nucleotide, hsa-mir-376a-1_9g could repress *Napb* in mouse (Supplementary Figure S9F).

To summarize, hsa-mir-376a-1_9g directly represses *GPR85* in human and monkey, and putatively in some primates, but not in mouse and those primates with non-conserved complementary sites of hsa-mir-376a-1_9g, which might contributes to the advanced functions and fast evolving of superior frontal gyrus in human. hsa-mir-376a-1_9g represses *NAPB* in human, monkey, and mouse because the complementary site of hsa-mir-376a-1_9g on mouse Napb only has one different nucleotide that forms a G-to-U pair with the A-to-I edited nucleotide in hsa-mir-376a-1_9g.

A previous study reported that there were multiple A-to-I editing sites in the miRNA cluster of miR-376, and the edited miR-376 could regulate a group of different target genes (Kawahara et al., 2007). Phosphoribosylpyrophosphate synthetase 1 (*PRPS1*) is one of the target genes of A-to-I edited miR-376a-5p (Kawahara et al., 2007). We examined the expression levels of *PRPS1* too. The expression of *PRPS1* was generally down-regulated in different gene expression profiles of ASD. For examples, *PRPS1* was significantly downregulated in STG (GSE64018), FC2 (GSE28521), and CE2 (GSE38322) (Supplementary Figures S10A–C and Supplementary Table S21). And *PRPS1* had non-significant downregulation trends in TC2 (GSE28521), OC (BA19) (GSE38322), CC (GSE62098), and cerebellum (CE1) (GSE28521) (Supplementary Figures S10D–G). In addition, *PRPS1* showed non-significant upregulation trends in DLPFC (GSE102741), and BT (GSE28475) (Supplementary Figures S10H, I).

## 4. Discussion

Our results indicated that the editing level of hsa-mir-376a-1_9_A_g was significantly increased in the ASD-SFG samples when compared to normal controls. The expression levels of *GPR85* and *NAPB* in the ASD-SFG sample were potentially repressed by the edited hsa-mir-376a-5p, which resulted in the significant down-regulation of *GPR85* and *NAPB* in some of the brain regions of ASD patients (Figures 5C–G). *GPR85* is an orphan receptor that regulates diverse behaviors including learning and memory, as well as neuronal and synaptic plasticity (Fujita-Jimbo et al., 2015). The mutated *GPR85* in ASD patients interferes with the formation of dendrites, and may become one of the pathogenesis molecules of ASD through the related NLGN-PSD-95 receptor complex (Fujita-Jimbo et al., 2015). Mutated *GPR85* can cause endoplasmic reticulum (ER) stress and interfere with the dendritic formation of hippocampal neurons (Fujita-Jimbo et al., 2015). *GPR85* is abundantly expressed in brain structures that exhibit high levels of plasticity, and *GPR85* is involved in determining the size of the brain, regulating diverse behaviors, and may cause schizophrenia (Matsumoto et al., 2008). *NAPB* plays a role in the fusion of vesicles in presynaptic membranes (Lisboa et al., 2019). *NAPB* is a new type of SNARE-associated protein, and the importance of SNARE complex in the development of epilepsy is recognized in the identification of pathogenic variants of *NAPB* (Conroy et al., 2016). In summary, the reduced expression of *GPR85* and *NAPB* may contribute to the abnormal neuronal development in ASD patients.

*PRPS1* is a reported target of hsa-mir-376a-1_9g. The mutations of *PRPS1* lead to some neurodevelopmental diseases too, including PRS-I superactivity, Charcot-Marie-Tooth disease-5 (CMTX5, or Rosenberg-Chutorian syndrome), Arts syndrome, and X-linked nonsyndromic sensorineural deafness (DFN2) (Brouwer et al., 2010). Overproduction of uric acid, intellectual disability, ataxia, hypotonia, and hearing impairment are the main manifestations of PRS-I superactive patients (Brouwer et al., 2010). Peripheral neuropathy, early-onset hearing loss and optic atrophy are characteristic phenotypes of CMTX5 (Rosenberg and Chutorian, 1967; Kim et al., 2007). Intellectual disability, early-onset hypotonia, ataxia, delayed motor development, hearing impairment, and optic atrophy are characteristic manifestations of patients with Art syndrome (Arts et al., 1993). Currently, replacement of purines by supplementing S-adenosylmethionine (SAM) appears to improve the condition of patients with Art syndrome (Mittal et al., 2015). The symptoms of *PRPS1* deficiency can be alleviated by supplementing SAM, and SAM supplementation is a new way to treat and intervene *PRPS1* deficiency (Mittal et al., 2015). An isolated symptom in DFN2 is X-linked postlingual nonsyndromic hearing loss (Liu et al., 2010). These suggest that increased editing level of hsa-mir-376a-1_9_A_g may result in the downregulation of *PRPS1* in different brain regions of ASD patients, which joins the pathology of ASD as well.

We compared the editing levels of the identified M/E sites for ASD-SFG brain samples and normal controls under the age of 10 years. We identified 117 M/E sites with significantly different editing levels, which was larger than that obtained when comparing all ASD-SFG to all SFG samples. And hsa-mir-376a-1_9_A_g appeared in both results. When being compared to the results for all samples, the editing level of hsa-mir-376a-1_9_A_g showed a more severe increase in ASD-SFG under 10 years than in SFG samples under 10 years. By studying the expression patterns of ADARs, we found that the expression level of *ADAR1* was significantly up-regulated in brains of postmortem ASD patients, cerebellum of ASD patients and occipital cortex of ASD patients compared to normal controls (Figures 4A–C, respectively). And *ADAR1* showed clearer increased expression in ASD samples under 10 year old compared to normal controls of the same ages (Figures 4J, K), which was consistent with the increase of A-to-I editing levels of miRNA editing sites, including hsa-mir-376a-1_9_A_g, in ASD-SFG samples under 10 years. These results suggest that the increased editing level of hsa-mir-376a-1_9_A_g in ASD-SFG samples under 10 year old might play a role in the initiation or progression of ASD, and the increased expression of *ADAR1* in ASD samples under 10 year old contributes to the increased miRNA A-to-I editing in ASD.

Furthermore, we found significant correlation between the editing levels of many editing sites, including hsa-mir-376a-1_9_A_g, and the ages of normal people, which disappears in ASD patients. This again indicates disturbed miRNA editing patterns in ASD and suggests a potential role miRNA editing in the etiology of ASD.

Our results suggest that novel therapies of ASD might be designed by either overexpressing *GPR85*/*NAPB* or repressing editing level of hsa-mir-376a-1_9_A_g. And the editing level of hsa-mir-376a-1_9_A_g in ASD is significantly increased, which might be used to develop new diagnostic methods for ASD.

## Data availability statement

The original contributions presented in the study are included in the article/Supplementary material, further inquiries can be directed to the corresponding author.

## Author contributions

YZ conceived and designed the research. XW, HY, HL, AS, SW, WX, NZ, SG, HD, GZ, JY, and YZ analyzed the data and organized the results. XW, SW, WX, ZQ, and HS performed the luciferase experiments. XW and YZ wrote the original manuscript. HY and YZ revised the manuscript. All authors contributed to the article and approved the submitted version.

## Supplementary material

Supplementary Figure S1 The distribution of different types of editing sites in miRNAs.

Supplementary Figure S2 The details of 24 identified A-to-I editing sites in miRNAs.

Supplementary Figure S3 The details of 5 identified C-to-U editing sites in miRNAs.

Supplementary Figure S4 The identified SNP sites in miRNAs.

Supplementary Figure S5 The expression of *ADAR1, ADAR2* and *ADAR3* in different brain regions of ASD patients and normal controls.

Supplementary Figure S6 The expression of *ADAR1, ADAR2* and *ADAR3* in dorsolateral prefrontal cortex of ASD patients and normal controls before 10 year old.

Supplementary Figure S7 The expression of *GPR85* in different brain regions of ASD patients and normal controls.

Supplementary Figure S8 The expression of *NAPB* in different brain regions of ASD patients and normal controls.

Supplementary Figure S9 Conservation and validation of *GPR85* and *NAPB* as targets of A-to-I edited miR-376a-5p.

Supplementary Figure S10 The expression of *PRPS1* in different brain regions of ASD patients and normal controls.

Supplementary Table S1 The 131 sRNA-seq profiles of ASD patients and normal controls analyzed in this study (Supplementary Table S1.1). The 8 gene expression profiles of different regions of brains in ASD patients and normal controls. (Supplementary Table S1.2). The 11 PAR-CLIP sequencing profiles used to predict targets for original and edited miRNAs (Supplementary Table S1.3).

Supplementary Table S2 The 834 editing sites that have significant editing levels in the selected sRNA-seq profiles (Supplementary Table S2.1). The 155 editing sites whose editing levels are significantly correlated with ages of the individuals in SFG (Supplementary Table S2.2). The 13 editing sites whose editing levels are significantly correlated with ages of the individuals in ASD-SFG (Supplementary Table S2.3). The 24 A-to-I editing sites identified in the selected samples (Supplementary Table S2.4). The 5 C-to-U editing sites identified in the selected samples (Supplementary Table S2.5). The 8 SNPs in miRNAs identified in the selected samples (Supplementary Table S2.6).

Supplementary Table S3 The 70 editing sites whose editing levels in SFG and ASD-SFG samples are significantly different.

Supplementary Table S4 The 117 editing sites whose editing levels in SFG and ASD-SFG samples under the age of ten are significantly different.

Supplementary Table S5 The targets of selected miRNA hsa-miR-376a-5p (Supplementary Table S5.1). The targets of selected A-to-I edited miRNA hsa-mir-376a-1_9g (Supplementary Table S5.2).

Supplementary Table S6 Source data of Figure 1.

Supplementary Table S7 Source data of Figure 2.

Supplementary Table S8 Source data of Figure 3.

Supplementary Table S9 Source data of Figure 4.

Supplementary Table S10 Source data of Figure 5.

Supplementary Table S11 Source data of Figure 6.

Supplementary Table S12 Source data of Supplementary Figure S1.

Supplementary Table S13 Source data of Supplementary Figure S2.

Supplementary Table S14 Source data of Supplementary Figure S3.

Supplementary Table S15 Source data of Supplementary Figure S4.

Supplementary Table S16 Source data of Supplementary Figure S5.

Supplementary Table S17 Source data of Supplementary Figure S6.

Supplementary Table S18 Source data of Supplementary Figure S7.

Supplementary Table S19 Source data of Supplementary Figure S8.

Supplementary Table S20 Source data of Supplementary Figure S9.

Supplementary Table S21 Source data of Supplementary Figure S10.
