# Supplementary material for: Characterizing microRNA editing and mutation sites in Autism Spectrum Disorder
Source: Front Mol Neurosci. 2023 Jan 20;15:1105278. doi: 10.3389/fnmol.2022.1105278 (PMC9895120; doi:10.3389/fnmol.2022.1105278)
Supplement: Supplementary Figure S1 — The distribution of different types of editing sites in miRNAs. [file Data_Sheet_1.zip › Data Sheet 1/ASD-SupplementaryData-v4.pdf]

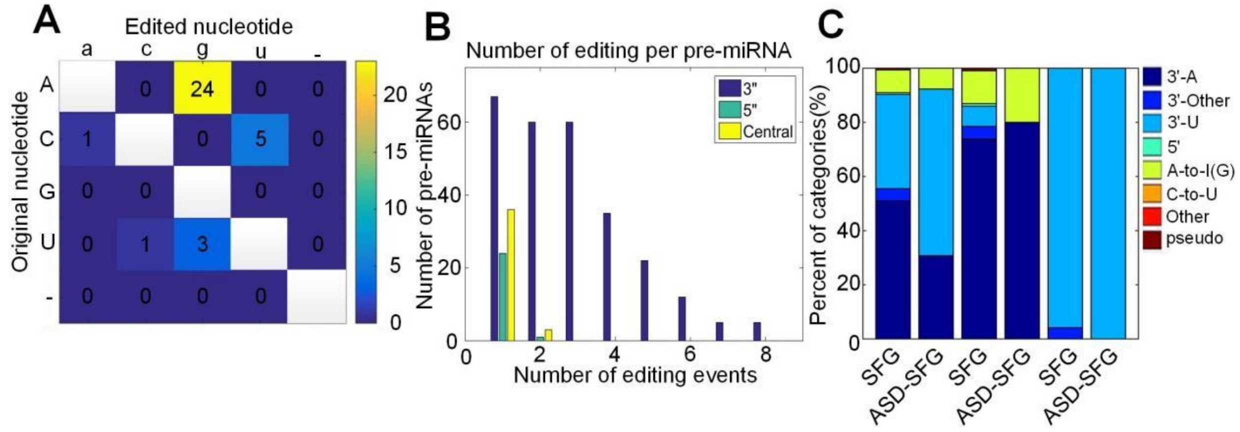

Supplementary Figure S1 The distribution of different types of editing sites in miRNAs. (**A**) The numbers of different types of editing events that do not happen at the 5' or 3' end of mature miRNAs. (**B**) The distribution of the numbers of pre-miRNAs with different numbers of 5'-, 3'- and Central editing sites, i.e. editing sites that do not happen at the ends of mature miRNAs. (**C**) The percentages of different types of miRNA editing sites whose editing levels are significantly correlated with the ages of individuals in SFG and ASD-SFG samples. The values in the parenthesis are the Kullback-Leibler divergences between the distributions of SFG and ASD-SFG. The source data are provided in Supplementary Table S12.

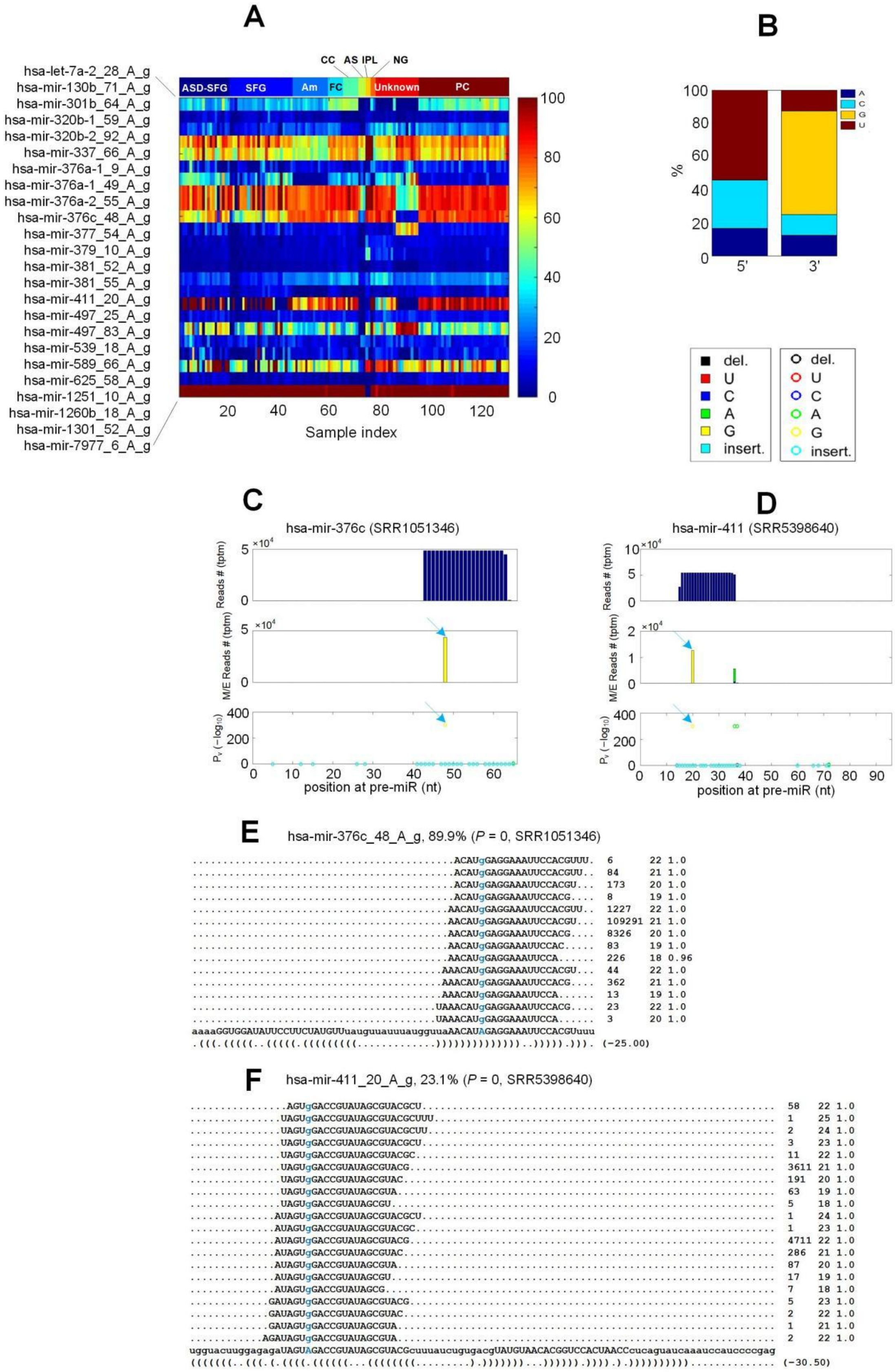

A
B
C
D
E
F
hsa-let-7a-2_28_A_g
hsa-mir-130b_71_A_g
hsa-mir-301b_64_A_g
hsa-mir-320b-1_59_A_g
hsa-mir-320b-2_92_A_g
hsa-mir-337_66_A_g
hsa-mir-376a-1_9_A_g
hsa-mir-376a-1_49_A_g
hsa-mir-376a-2_55_A_g
hsa-mir-376c_48_A_g
hsa-mir-377_54_A_g
hsa-mir-379_10_A_g
hsa-mir-381_52_A_g
hsa-mir-381_55_A_g
hsa-mir-411_20_A_g
hsa-mir-497_25_A_g
hsa-mir-497_83_A_g
hsa-mir-539_18_A_g
hsa-mir-589_66_A_g
hsa-mir-625_58_A_g
hsa-mir-1251_10_A_g
hsa-mir-1260b_18_A_g
hsa-mir-1301_52_A_g
hsa-mir-7977_6_A_g
CC
AS
IPL
NG
ASD-SFG
SFG
Am
FC
Unknown
PC
Sample index
%
5'
3'
A
C
G
U
del.
insert.
hsa-mir-376c (SRR1051346)
hsa-mir-411 (SRR5398640)
Reads # (tptm)
M/E Reads # (tptm)
P_v (−log10)
position at pre-miR (nt)
hsa-mir-376c_48_A_g, 89.9% (P = 0, SRR1051346)
aaaaGGUGGAUAUUCCUUCUAUGUUuauguuauuuaugguuaAACAUAGAGGAAAUUCCACGUuuu
(−25.00)
hsa-mir-411_20_A_g, 23.1% (P = 0, SRR5398640)
ugguacuuggagagaUAGUAGACCGUAUAGCGUACGcuuuaucugugacgUAUGUAACACGGUCCACUAACCcucaguaucaaauccaucccgag
(−30.50)

Supplementary Figure S2 The details of 24 identified A-to-I editing sites in miRNAs. (**A**) The editing levels of the 24 A-to-I editing sites in the 131 selected brain data sets. The 131 samples include 20 superior frontal gyrus samples of postmortem ASD patients (ASD-SFG), 25 superior frontal gyrus samples of normal controls (SFG), 14 amygdalae samples of normal controls (Am), 6 frontal cortex of normal controls (FC), 6 corpus callosum samples of normal controls (CC), 2 inferior parietal lobe samples of normal controls (IPL), 2 temporal neocortex gray matter samples of normal controls (NG), 3 astrocyte cell lines of normal controls (As), 36 prefrontal cortex of normal controls (PC) and 17 unknown brain regions of normal controls (Unknown). (**B**) The percentages of nucleotides beside the 24 A-to-I editing sites. (**C**) The MiRME map of hsa-mir-376c in one of the inferior parietal lobe normal samples (SRR1051346). The upper panel shows the total numbers of reads that cover each nucleotide of the pre-miRNA. The central panel shows the numbers of M/E reads at each position of the pre-miRNA. And the lower panel givens multiple test corrected *P*-values (-log10 scaled) of the corresponding mutation/editing sites shown in the central panel. (**D**) The MiRME map of hsa-mir-411 in one of the amygdala normal samples (SRR5398640). (**E**) The details of hsa-mir-376c_48_A_g in SRR1051346. (**F**) The details of hsa-mir-411_20_A_g in SRR5398640. In Part (E) and (F), the edited nucleotides are shown in bold face. The source data are provided in Supplementary Table S13.

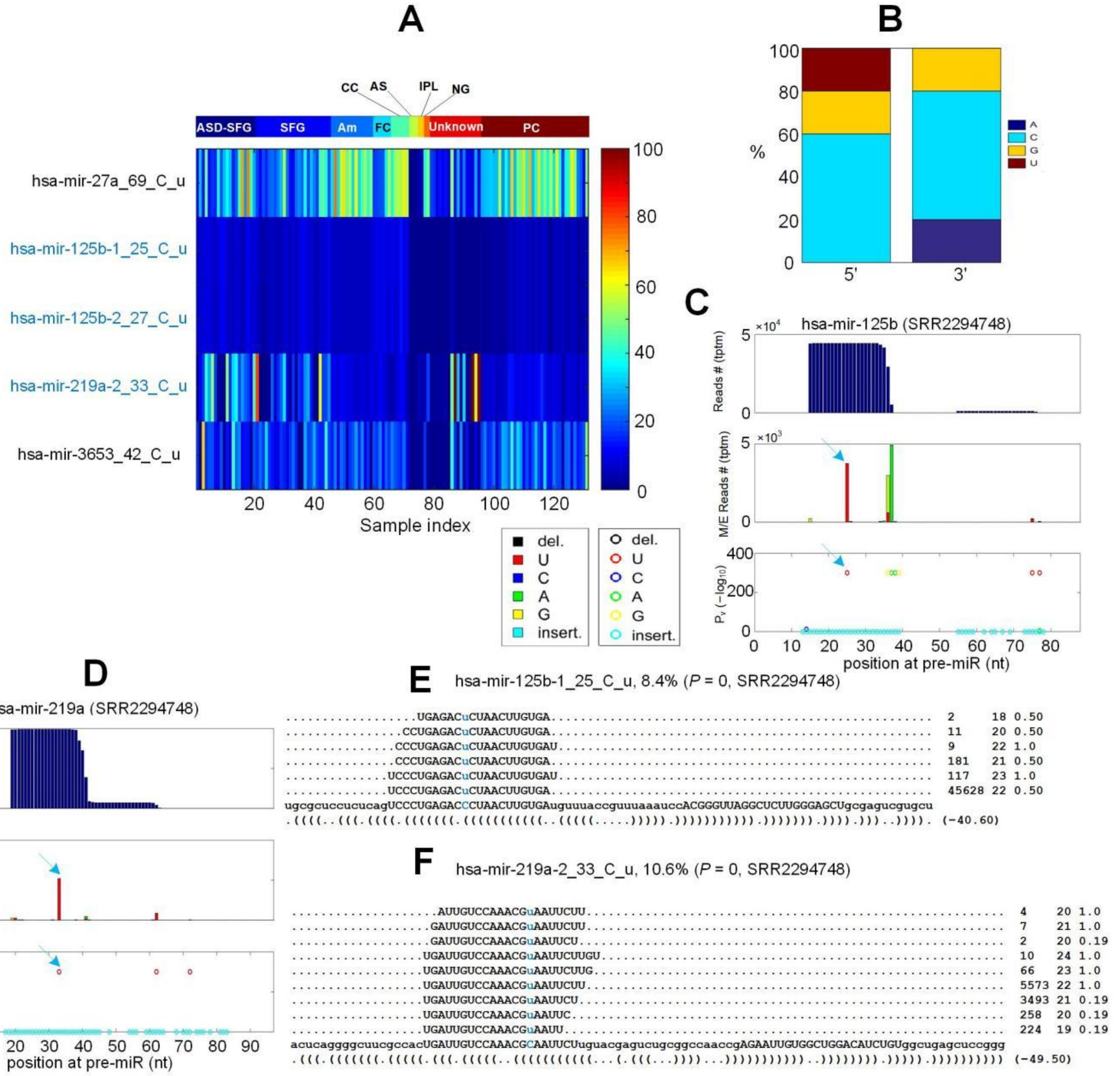

Supplementary Figure S3 The details of 5 identified C-to-U editing sites in miRNAs. (**A**) The editing levels of the 5 identified C-to-U editing sites in the 131 sRNA-seq profiles. The numbers of samples from different groups are the same as those in Extended Data Fig. 2a. The 5 sites with blue names are conserved C-to-U editing sites in primates. (**B**) The percentages of nucleotides beside these 5 C-to-U editing sites. (**C**) The MiRME map of hsa-mir-125b in one of the normal corpus callosum (CC) samples (SRR2294748). (**D**) The MiRME map of hsa-mir-219a-2 in one of the normal corpus callosum (CC) samples (SRR2294748). (**E**) The details of hsa-mir-125b-1_25_C_u in SRR2294748. (**F**) The details of hsa-mir-219a-2_33_C_u in SRR2294748. The source data are provided in Supplementary Table S14.

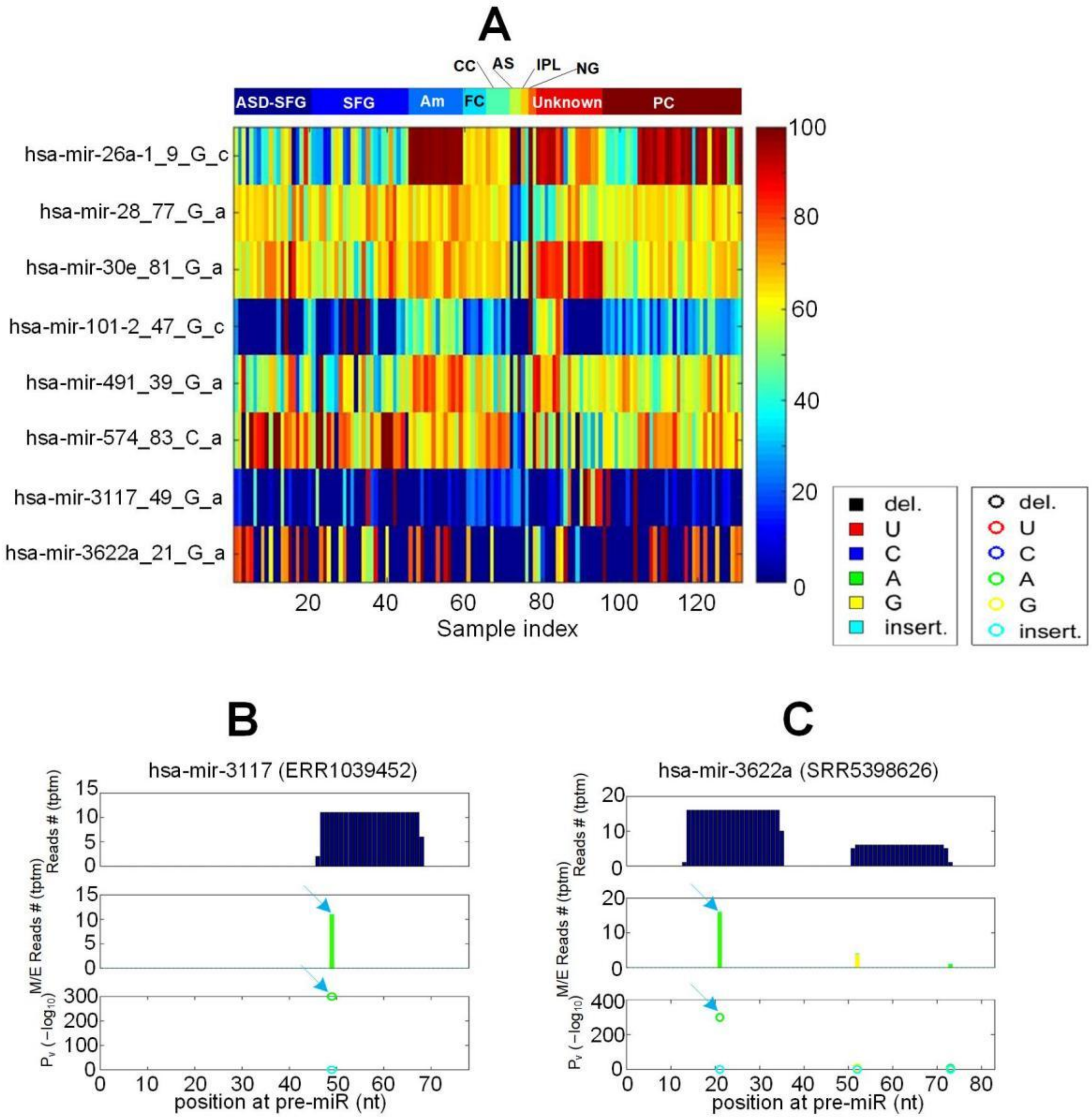

D hsa-mir-3117_49_G_a~rs12402181, 100.0% (*P* = 0, ERR1039452)

```
..............................................UAaGACUCAUAUAGUGCCAGGU.........   6 22 1.0
..............................................UAaGACUCAUAUAGUGCCAGG..........   3 21 1.0
.............................................AUAaGACUCAUAUAGUGCCAGG..........   2 22 1.0
cccuaaagggccAGACACUAUACGAGUCAUAUaagggaaggcauuAUAGGACUCAUAUAGUGCCAGguguuuguggg
(((((((.(((.(.(((((((.(((((.(((((.(.....).))))).))))).))))))).).))).))))).)))  (-30.10)
```

E hsa-mir-3622a_21_G_a~rs66683138, 100.0% (*P* = 0, SRR5398626)

```
.............CAGGCACaGGAGCUCAGGUGAG................................................ 13 22 1.0
.............CAGGCACaGGAGCUCAGGUGA................................................. 5  21 1.0
............ACAGGCACaGGAGCUCAGGUGA................................................. 2  22 1.0
aauagagggugcaCAGGCACGGGAGCUCAGGUGAGgcagggagcugagcUCACCUGACCUCCCAUGCCUGUgcacccucuauu
.((((((((((((((((.(((((.(((((((((.(((....)))..))))))))).))))).))))))))))))))))).  (-68.20)
```

Supplementary Figure S4 The identified SNP sites in miRNAs. (**A**) The editing levels of 8 SNP sites in the 131 selected brain data sets. (**B**) The MiRME map of hsa-mir-3117 in one the normal Unknown Region (Unknown) samples (ERR1039452). (**C**) The MiRME map of hsa-mir-3622a in one the normal Amygdalae (Am) samples (SRR5398626). (**D**) The details of hsa-mir-3117_49_G_a, i.e., rs12402181, in ERR1039452. (**E**) The details of hsa-mir-3622a_21_G_a, i.e., rs66683138, in SRR5398626. The source data are provided in Supplementary Table S15.

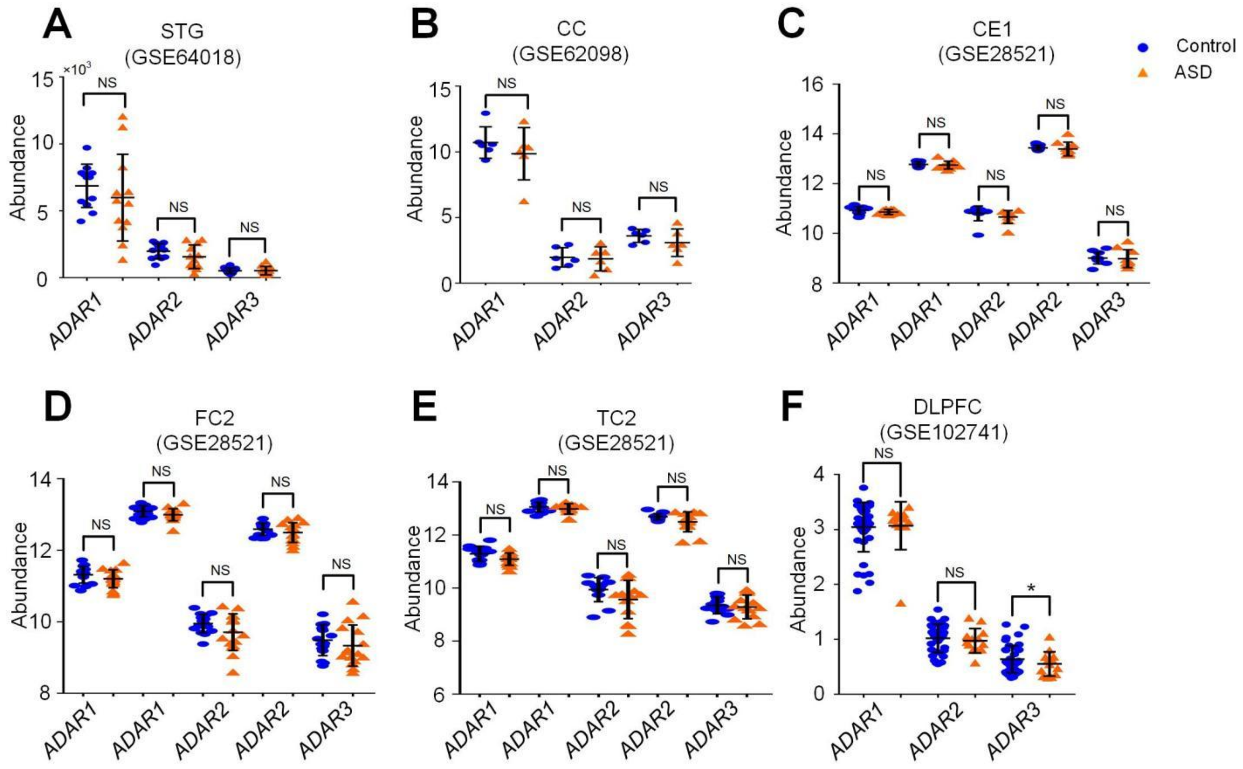

Supplementary Figure S5 The expression of *ADAR1*, *ADAR2* and *ADAR3* in different brain regions of ASD patients and normal controls. (**A**) The abundance of *ADAR1*, *ADAR2* and *ADAR3* in superior temporal gyrus (STG) samples of Normal controls (NC) and ASD patients (ASD) (GSE64018). The *P*-value was calculated with the edger package. (**B**) The abundance of *ADAR1*, *ADAR2* and *ADAR3* in corpus callosum (CC) samples of ASD patients (ASD) and normal controls (NC) (GSE62098). The *P*-value was calculated with the edger package. (**C**) The abundance of *ADAR1* (ILMN_2320964), *ADAR1* (ILMN_1776777), *ADAR2* (ILMN_2319326), *ADAR2* (ILMN_1679797) and *ADAR3* (ILMN_1749493) in cerebellum (CE1) samples of ASD patients (ASD) and normal controls (NC) (GSE28521). The *P*-value was calculated with the limma package. (**D**) The abundance of *ADAR1* (ILMN_2320964), *ADAR1* (ILMN_1776777), *ADAR2* (ILMN_2319326), *ADAR2* (ILMN_1679797) and *ADAR3* (ILMN_1749493) in Frontal cortex (FC2) samples of Normal controls (NC) and ASD patients (ASD) (GSE28521). The *P*-value was calculated with the limma package. (**E**) The abundance of *ADAR1* (ILMN_2320964), *ADAR1* (ILMN_1776777), *ADAR2* (ILMN_2319326), *ADAR2* (ILMN_1679797) and *ADAR3* (ILMN_1749493) in temporal cortex (TC2) samples of ASD patients (ASD) and normal controls (NC) (GSE28521). The *P*-value was calculated with the limma package. (**F**) The abundance of *ADAR1*, *ADAR2* and *ADAR3* in dorsolateral prefrontal cortex (DLPFC) samples of ASD patients (ASD) and normal controls (NC) (GSE102741). The *P*-value was calculated with the limma package. The source data are provided in Supplementary Table S16. *: $P < 0.05$; NS: not significant.

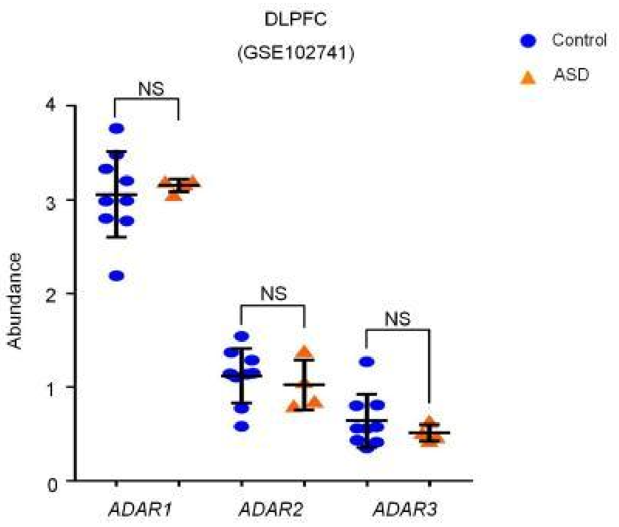

Supplementary Figure S6 The expression of *ADAR1*, *ADAR2* and *ADAR3* in dorsolateral prefrontal cortex of ASD patients and normal controls before 10 years old. The abundance of *ADAR1*, *ADAR2* and *ADAR3* in dorsolateral prefrontal cortex (DLPFC) samples of Normal controls (NC) and ASD patients (ASD) (GSE102741). The *P*-value was calculated with the two-tailed *t*-test. The source data are provided in Supplementary Table S17. NS: not significant.

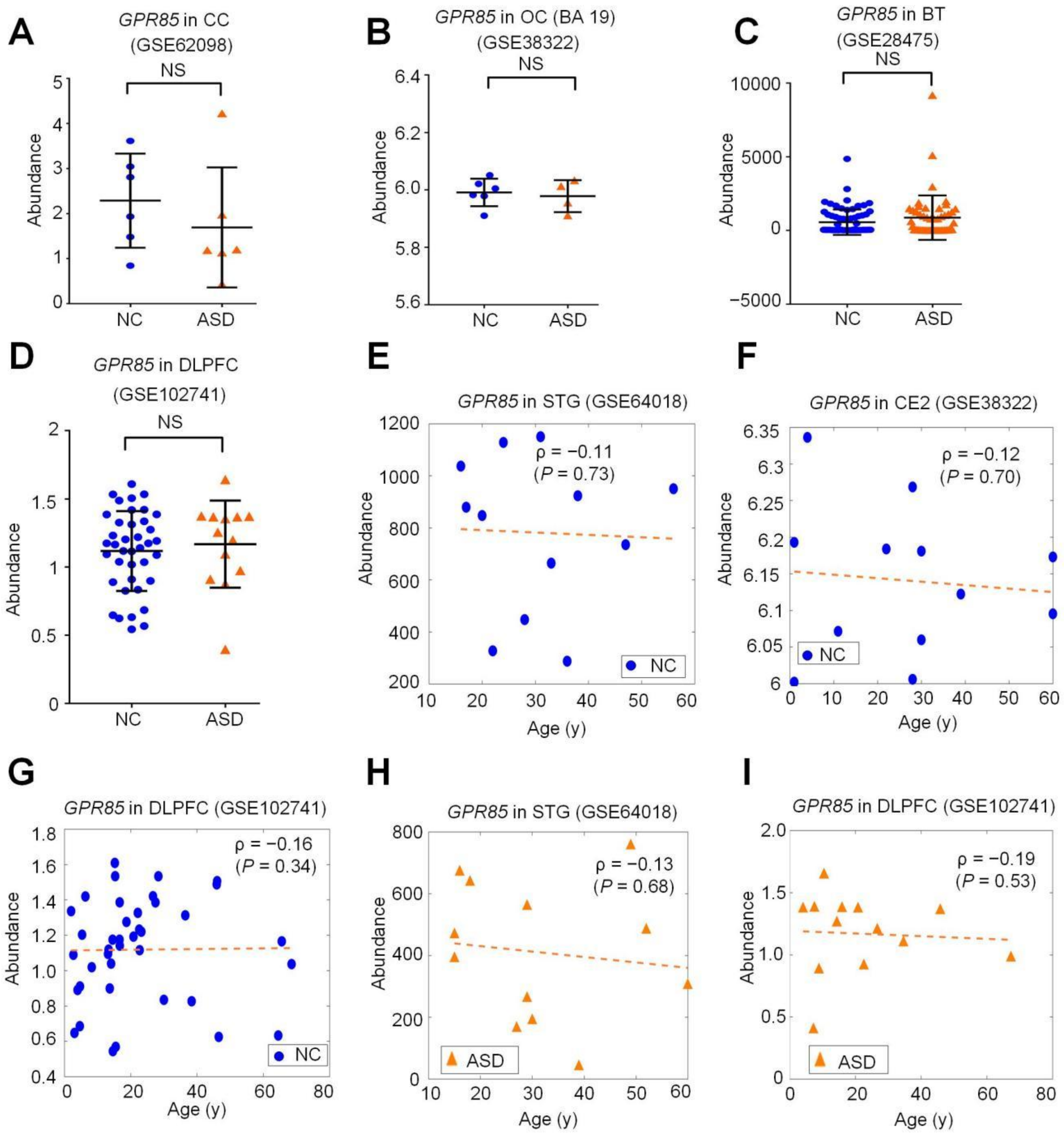

Supplementary Figure S7 The expression of *GPR85* in different brain regions of ASD patients and normal controls. (**A**) The abundance of *GPR85* in corpus callosum (CC) samples of normal controls (NC) and ASD patients (ASD) (GSE62098). The *P*-value was calculated with the limma package. (**B**) The abundance of *GPR85* in occipital cortex (OC) (BA 19) samples of normal controls (NC) and ASD patients (ASD) (GSE38322). The *P*-value was calculated with the limma package. (**C**) The abundance of *GPR85* in postmortem brain tissue (BT) samples of normal controls (NC) and ASD patients (ASD) (GSE28475). The *P*-value was calculated with the limma package. (**D**) The abundance of *GPR85* in dorsolateral prefrontal cortex (DLPFC) samples of normal controls (NC) and ASD patients (ASD) (GSE102741). The *P*-value was calculated with

the limma package. (**E**) The Spearman correlation (ρ) between the ages of superior temporal gyrus (STG) samples of normal controls (NC) and the abundances of *GPR85* in GSE64018. (**F**) The Spearman correlation (ρ) between the ages of cerebellum (CE2) samples of normal controls (NC) and the abundances of *GPR85* in GSE38322. (**G**) The Spearman correlation between the ages of DLPFC samples of normal controls (NC) and the abundances of *GPR85* in GSE102741. (**H**) The Spearman correlation between the ages of STG samples of ASD patients (ASD) and the abundances of *GPR85* in GSE64018. (**I**) The Spearman correlation between the ages of the DLPFC samples of ASD patients (ASD) and the abundances of *GPR85* in GSE102741. The source data are provided in Supplementary Table S18. NS: not significant.

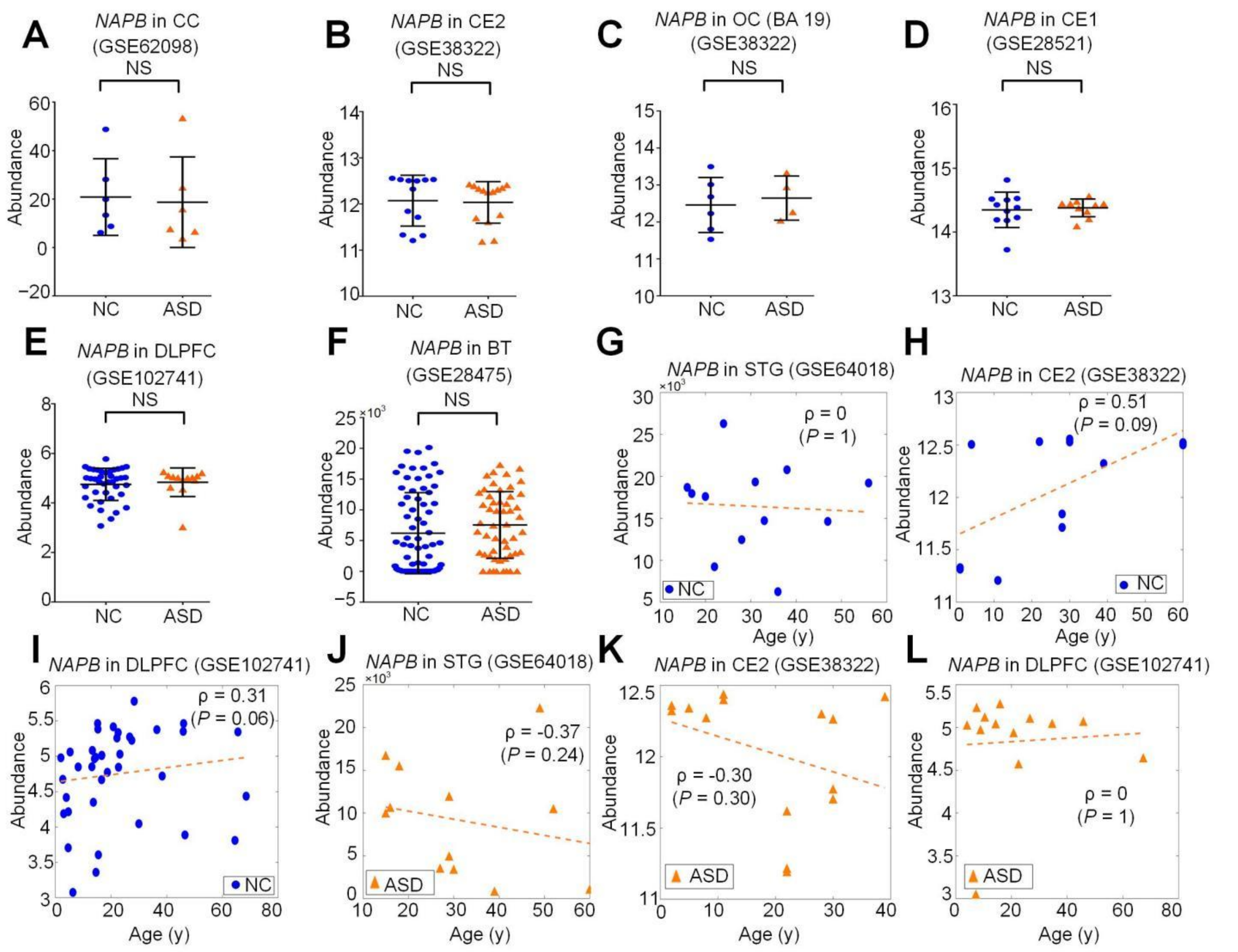

Supplementary Figure S8 The expression of *NAPB* in different brain regions of ASD patients and normal controls. (**A**) The abundance of *NAPB* in corpus callosum (CC) samples of ASD patients (ASD) and normal controls (NC) (GSE62098). The *P*-value was calculated with the edger package. (**B**) The abundance of *NAPB* in cerebellum (CE2) samples of Normal controls (NC) and ASD patients (ASD) (GSE38322). The *P*-value was calculated with the limma package. (**C**) The abundance of *NAPB* in occipital cortex (OC) (BA 19) samples of ASD patients (ASD) and normal controls (NC) (GSE38322). The *P*-value was calculated with the limma package. (**D**) The abundance of *NAPB* in cerebellum (CE1) samples of ASD patients (ASD) and normal controls (NC) (GSE28521). The *P*-value was calculated with the limma package. (**E**) The abundance of *NAPB* in dorsolateral prefrontal cortex (DLPFC) samples of ASD patients (ASD) and normal controls (NC) (GSE102741). The *P*-value was calculated with the limma package. (**F**) The abundance of *NAPB* in postmortem brain tissue (BT) samples of ASD patients (ASD) and normal controls (NC) (GSE28475). The *P*-value was calculated with the limma package. (**G**) The Spearman correlation (ρ) between the ages of STG samples of normal controls (NC) and the abundances of *NAPB* in GSE64018. (**H**) The Spearman correlation (ρ) between the ages of cerebellum (CE2) samples of normal controls (NC) and the abundances of *NAPB* in GSE38322. (**I**) The Spearman correlation between the ages of DLPFC samples of normal controls (NC) and the abundances of *NAPB* in GSE102741. (**J**) The Spearman correlation between the ages of STG samples of ASD patients (ASD) and the abundances of *NAPB* in GSE64018. (**K**) The Spearman correlation between the ages of cerebellum (CE2) samples of ASD patients (ASD) and the

abundances of *NAPB* in GSE38322. (**L**) The Spearman correlation between the ages of DLPFC samples of ASD patients (ASD) and the abundances of VAPB in GSE102741. The source data are provided in Supplementary Table S19. NS: not significant.

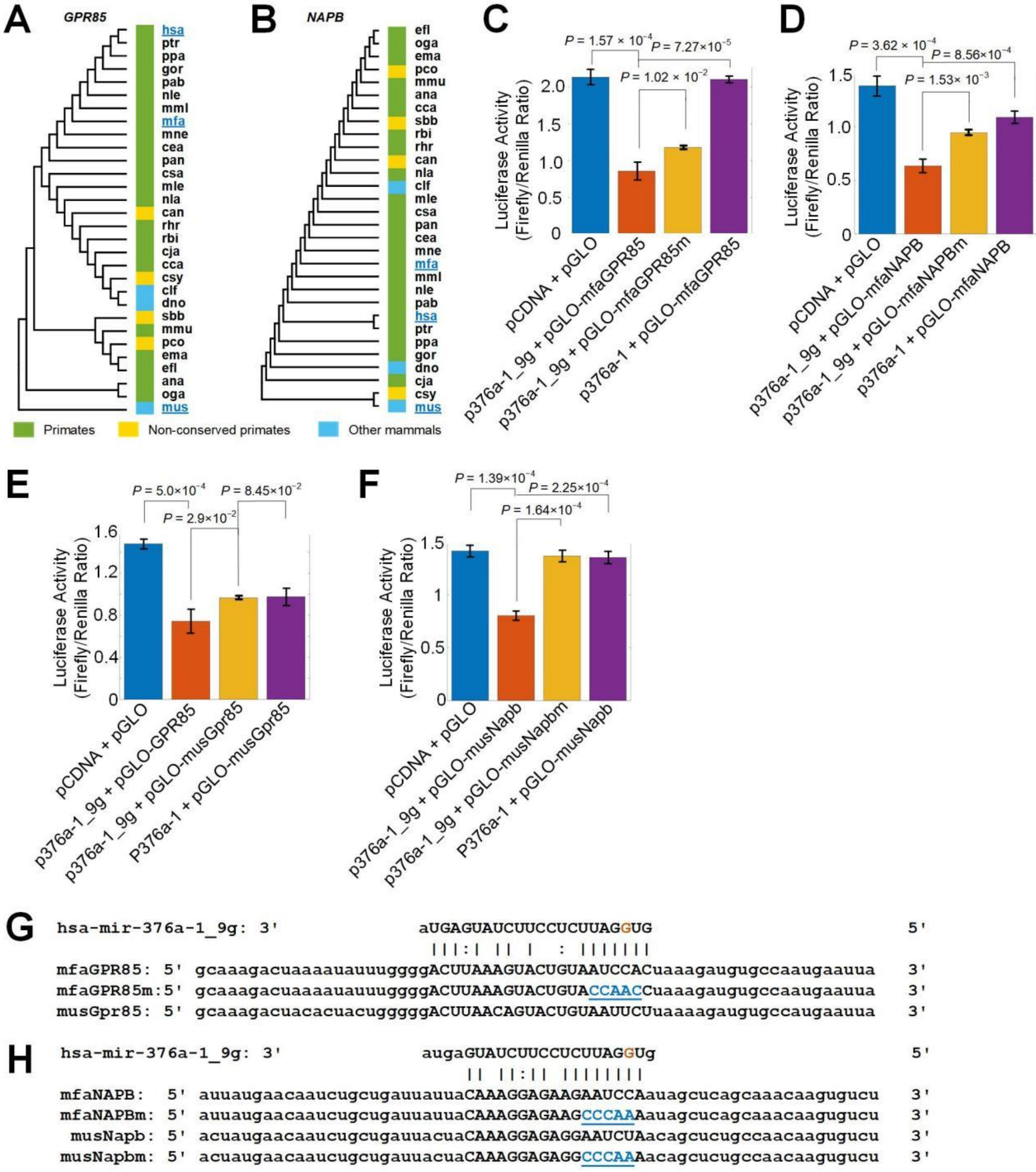

Supplementary Figure S9 Conservation and validation of *GPR85* and *NAPB* as targets of A-to-I edited miR-376a-5p. (**A**) Phylogenetic tree of A-to-I edited miR-376a-5p complementary sites on *GPR85* in 30 mammals. The abbreviations of the 30 species are listed in Supplementary Table S18. The three species with blue names, i.e., human (hsa, *Homo sapiens*), monkey (mfa, *Macaca fascicularis*) and mouse (mus, *Mus musculus*) were selected for luciferase experiments. (**B**) Phylogenetic tree of A-to-I edited miR-376a-5p complementary sites on *NAPB* in 30 mammals. Legend are the same as those of Part (G). (**C**) The luciferase activities when co-transfecting a pGLO plasmid of 3' UTRs of monkey *GPR85* in Part (A) and a pCDNA plasmid containing original pre-hsa-mir-376a (p376a) or pre-hsa-mir-376a-1_9g (p376a-1_9g), respectively. (**D**) The

luciferase activities when co-transfecting a pGLO plasmid of 3' UTRs of monkey *NAPB* in Part (B) and a pCDNA plasmid containing original pre-hsa-mir-376a (p376a) or pre-hsa-mir-376a-1_9g (p376a-1_9g), respectively. (**E**) The luciferase activities when co-transfecting a pGLO plasmid of 3' UTRs of mouse *Gpr85* in Part (A) and a pCDNA plasmid containing original pre-hsa-mir-376a (p376a) or pre-hsa-mir-376a-1_9g (p376a-1_9g), respectively. (**F**) The luciferase activities when co-transfecting a pGLO plasmid of 3' UTRs of mouse *Napb* in Part (B) and a pCDNA plasmid containing original pre-hsa-mir-376a (p376a) or pre-hsa-mir-376a-1_9g (p376a-1_9g), respectively. (**G**) The regions of monkey *GPR85* (mfaGPR85), mutated monkey *GPR85* (mfaGPR85m) and mouse *Gpr85* (musGpr85) with A-to-I edited miR-376a-5p complementary sites. (**H**) The regions of monkey *NAPB* and mouse *NAPB* with A-to-I edited miR-376a-5p complementary sites. In Part (C) to (F), the values shown are mean values ± SDs. *P*-values were based on two-tailed *t*-tests. The source data are available in Supplementary Table S20.

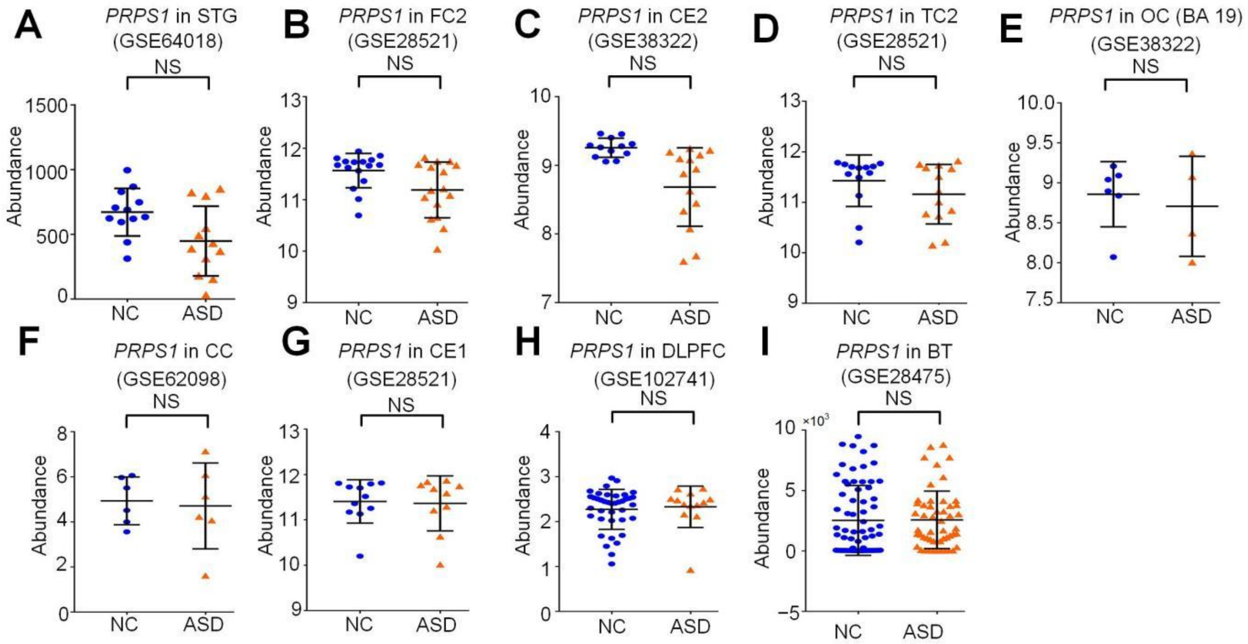

Supplementary Figure S10 The expression of *PRPS1* in different brain regions of ASD patients and normal controls. (**A**) The abundance of *PRPS1* in superior temporal gyrus (STG) samples of Normal controls (NC) and ASD patients (ASD) (GSE64018). The *P*-value was calculated with the edger package. (**B**) The abundance of *PRPS1* in Frontal cortex (FC2) samples of ASD patients (ASD) and normal controls (NC) (GSE28521). The *P*-value was calculated with the limma package. (**C**) The abundance of *PRPS1* in cerebellum (CE2) samples of Normal controls (NC) and ASD patients (ASD) (GSE38322). The *P*-value was calculated with the limma package. (**D**) The abundance of *PRPS1* in temporal cortex (TC2) samples of ASD patients (ASD) and normal controls (NC) (GSE28521). The *P*-value was calculated with the limma package. (**E**) The abundance of *PRPS1* in occipital cortex (OC) (BA 19) samples of ASD patients (ASD) and normal controls (NC) (GSE38322). The *P*-value was calculated with the limma package. (**F**) The abundance of *PRPS1* in corpus callosum (CC) samples of ASD patients (ASD) and normal controls (NC) (GSE62098). The *P*-value was calculated with the edger package. (**G**) The abundance of *PRPS1* in cerebellum (CE1) samples of ASD patients (ASD) and normal controls (NC) (GSE28521). The *P*-value was calculated with the limma package. (**H**) The abundance of *PRPS1* in dorsolateral prefrontal cortex (DLPFC) samples of ASD patients (ASD) and normal controls (NC) (GSE102741). The *P*-value was calculated with the limma package. (**I**) The abundance of *PRPS1* in postmortem brain tissue (BT) samples of ASD patients (ASD) and normal controls (NC) (GSE28475). The *P*-value was calculated with the limma package. The source data are provided in Supplementary Table S21. NS: not significant.
